# Do high-protein diets have the potential to reduce gut barrier function in a sex-dependent manner?

**DOI:** 10.1007/s00394-024-03407-w

**Published:** 2024-04-25

**Authors:** Daniel James, Carlos Poveda, Gemma E. Walton, J. Stephen Elmore, Brandon Linden, John Gibson, Bruce A. Griffin, M. Denise Robertson, Marie C. Lewis

**Affiliations:** 1 Department of Food and Nutritional Sciences, University of Reading, Whiteknights Campus, Reading, RG6 6DZ UK; 2 Department of Nutrition, Food & Exercise Sciences, University of Surrey, Guildford, GU2 7XH UK; 3 Food and Feed Innovations, Woodstock, Newcastle Rd, Woore, N Shropshire, CW3 95N UK

**Keywords:** Dietary protein, Gut microbiota, Sexual dimorphisms, In vitro gut systems, Microbial-derived metabolic end products

## Abstract

**Purpose:**

Impaired gut barrier function is associated with systemic inflammation and many chronic diseases. Undigested dietary proteins are fermented in the colon by the gut microbiota which produces nitrogenous metabolites shown to reduce barrier function in vitro. With growing evidence of sex-based differences in gut microbiotas, we determined whether there were sex by dietary protein interactions which could differentially impact barrier function *via* microbiota modification.

**Methods:**

Fermentation systems were inoculated with faeces from healthy males (*n* = 5) and females (*n* = 5) and supplemented with 0.9 g of non-hydrolysed proteins sourced from whey, fish, milk, soya, egg, pea, or mycoprotein. Microbial populations were quantified using fluorescence in situ hybridisation with flow cytometry. Metabolite concentrations were analysed using gas chromatography, solid phase microextraction coupled with gas chromatography-mass spectrometry and ELISA.

**Results:**

Increased protein availability resulted in increased proteolytic *Bacteroides* spp (*p* < 0.01) and *Clostridium coccoides* (*p* < 0.01), along with increased phenol (*p* < 0.01), *p*-cresol (*p* < 0.01), indole (*p* = 0.018) and ammonia (*p* < 0.01), varying by protein type. Counts of *Clostridium* cluster IX (*p* = 0.03) and concentration of *p*-cresol (*p* = 0.025) increased in males, while females produced more ammonia (*p* = 0.02), irrespective of protein type. Further, we observed significant sex-protein interactions affecting bacterial populations and metabolites (*p* < 0.005).

**Conclusions:**

Our findings suggest that protein fermentation by the gut microbiota in vitro is influenced by both protein source and the donor’s sex. Should these results be confirmed through human studies, they could have major implications for developing dietary recommendations tailored by sex to prevent chronic illnesses.

**Supplementary Information:**

The online version contains supplementary material available at 10.1007/s00394-024-03407-w.

## Introduction

With the exception of its role in food sensitivity and food allergy, dietary protein is seldom viewed as having anything other than positive effects on health. Consequently, dietary guidelines encouraging populations to increase protein intake by up to 100%, in a bid to combat the effects of sarcopenia [1], have been made with little consideration given to safety in terms of potential adverse effects on gut, immune and metabolic health. Until recently, digestion and absorption of dietary protein was considered to be highly efficient. However, recent evidence suggests that up to 10% of consumed protein reaches the colon undigested in humans [2]. The colon is host to the greatest density of commensal bacteria in mammals [3], and is where the highest level of bacterial fermentation of undigested food products occurs. Undigested colonic dietary protein is fermented by specific components of the resident microbiota [4] and thus has the potential to drive the expansion of proteolytic populations at the expense of more beneficial groups such as bifidobacteria and lactobacilli. Work in this area is limited, but intervention studies using varying amounts of increased dietary protein and time have reported changes in colonic bacterial metabolism [5–7], with increased dietary protein intake being linked to adverse shifts in bacterial populations [8]. Such changes can result in increases in the production of microbial-derived negative end-point metabolites, including ammonia and phenolic compounds, which have been shown to reduce gut barrier function in vitro [9–12]. The gut wall provides a physical, selective barrier preventing potentially harmful products of gut bacteria and food digestion from entering blood circulation [13]. Reductions in this barrier function through inflammation, disruption, and impaired integrity of the gut lining, a condition often referred to as ‘leaky gut’, can result in increased passage of these products, such as lipopolysaccharide (LPS), into blood [13, 14]. This can cause chronic low-grade systemic inflammation and thus promote the development of chronic degenerative diseases of the liver and cardiovascular system [14, 15]. While leaky gut may have multifactorial causes, fermentation of excess dietary protein by colonic microbes could be an important contributor to the development of these chronic diseases.

Healthy adult females have been shown to express lower and more variable permeability in the gut barrier than males. Additionally, adult male gut barrier function appears more stable and is less sensitive to impairment caused by non-steroidal anti-inflammatories (NSAIDs) than females [16]. However, female gut barrier function is more resilient to shock states such as acidosis or hypoxia than that of males [17], but becomes less stable with age [18]. The cause of these differences is multifactorial and is thought to involve sex hormones, immunity and the gut microbiota [19–21]. For example, female microbiotas are significantly more diverse than those in males^,^ [22–24] and tend to have a lower abundance of Bacteroidetes, but higher proportions of Bacillota (Firmicutes) and Actinobacteria than male microbiotas [25, 26]. A greater diversity and high proportions of Bacillota can be indicative of a healthy microbiota [27], which might contribute to the greater efficiency observed in female barrier functionality. Microbiota sex differences also extend to lower taxonomic levels, with populations of *Prevotellaceae, Ruminococcaceae* [28], *Clostridiales* [22], and *Escherichia* [29] being lower in females than males. Specific microbial genera and species have been demonstrated to correlate with components of gastrointestinal immunity, including *Clostridium-*driven increases in intestinal mucosal regulatory T-cell (T_reg_) populations [30]. Higher levels of Clostridium populations in the male gut and the association between these microbes and increased levels of T_regs_, may contribute to the disproportionally lower incidence of inflammatory gut conditions observed in males compared to females.

Complex interactions occur between gut barrier integrity, immune function and the gut microbiota. Since there is sexual dimorphism in all three of these systems in healthy adults, it is likely that the detrimental effects of excess dietary proteins on microbial-derived metabolites associated with reduced barrier function could also be sexually dimorphic. To the best of our knowledge, this has not yet been explored and possible mechanisms remain undefined. However, significant sex-dependent responses to dietary interventions have previously been identified, including inulin, starch and the probiotic *Bifidobacteria lactis* in immune-associated protein expression in the gut of 28-day-old piglets [31]. This supports potential links between nutrition and sex-based differences in physiological responses.

Consumption of proteins originating from different sources has previously been shown to have differential effects on metabolic end-product production by bacterial populations in the gut [32, 33]. For example, rat caecal production of acetic acid was significantly higher following consumption of soya protein than casein [34], and lactic acid production was higher following fish meal consumption compared to casein and soy [32]. However, there are several inconsistencies across these studies, including where supplementing with proteins from the same source (soya) resulted in significantly different levels of acetic acid production by the gut microbiota [32, 34]. This could be explained by the protein being used in conjunction with different prebiotics, which could impact on microbiota metabolism differently to the microbial metabolic response to protein in the absence of prebiotics [34]. Variations observed might also be attributed to the disparities in intestinal microbiota across individuals and groups of experimental animals. In addition, results from rodent trials, although useful, may not directly reflect human responses to dietary proteins, since mice and rats have marked differences in gastrointestinal physiology to humans. This includes rodents having proportionally larger caecums along with smaller colons [35], and considerable differences in microbiota composition in comparison to humans. For example, only 25 of the 60 bacterial genera found in the murine microbiota are shared by humans [36]. Factors such as the strain of mouse [37] and animal housing environments [38, 39] can also cause significant differences in the composition of rodent microbiotas, as previously reviewed by Hugenholtz and de Vos [37].

Dietary proteins from animal and non-animal sources may also have distinct effects on the composition of gut bacterial populations. Xiao et al. demonstrated that dietary proteins derived from cereals were linked with proportional increases in *Bacteroides* spp. and *Phascolarctobacterium* spp. in vitro, in comparison to proteins from meat sources [33]. In addition to these findings, faecal microbial fermentation of chicken protein resulted in increased short chain fatty acid (SCFA) production compared to the other proteins tested [33]. However, data regarding changes to the production of phenolic compounds, which are known to reduce gut barrier function in vitro, were not reported. The purification processes for the meat-based test proteins were also not reported but can have important implications for digestibility and thus efficiency of transit to the colon. Nevertheless, differential effects of proteins from different origins on both microbiota composition and on the production of metabolic end-products suggests that protein source may be an important determinant regarding the potential links between increased dietary protein and impaired intestinal barrier function.

We hypothesised that excess non-hydrolysed dietary proteins from different sources would have differential effects on the composition of the microbiota, and on the production of bacterial-derived metabolic end-products, especially those which have been shown to have detrimental effects on intestinal barrier function. We further hypothesised that these differences would have sex-dependent origins. The aim of this work was to quantify the effects of increased availability of non-hydrolysed proteins from different sources on the gut microbiota and metabolite production using in vitro gut model systems. These were inoculated with stools from both male and female healthy human donors to explore any sexually dimorphic differences which may occur.

## Materials and methods

### Sample preparation for in vitro batch culture fermentation

Stool samples were collected from 10 (5 female; 5 male) healthy human donors (25–40 years) without any gastrointestinal disorders, who had not consumed antibiotics, laxatives, probiotics, or prebiotics for 2 months. Donors were not following vegetarian or vegan diets and were not actively consuming high protein diets or taking medications known to affect the gut microbiota. These were used to inoculate fermenters within 2 h of production and during this time, the faecal samples were kept under anaerobic conditions using anaerobic sachets (OxoidTM, AnaeroGen 2.5 L) (Thermo Scientific TM, 10,269,582) in collection jars. Anaerobic phosphate-buffered saline (0.1 M PBS, pH 7.4) was used to dilute the faecal sample to a 1:10 (w/v) ratio prior to homogenisation in a stomacher (Stomacher 400 Circulator Lab Blender, Seward, UK) for 2 min (460 paddle beats/min).

### Batch culture fermentations

Ten independent fermentations (5 female; 5 male) were completed, each with a different donor. The vessels were autoclaved prior to each experimental run and filled with 135 mL of basal media (peptone water (2 g/L), yeast extract (2 g/L), NaCl (0.1 g/L), K_2_HPO_4_ (0.04 g/L), KH_2_PO_4_ (0.04 g/L), MgSO_4_.7H_2_O (0.01 g/L), CaCl_2_.6H_2_O (0.01 g/L), NaHCO_3_ (2 g/L), Tween 80 (2 ml/L), haemin (0.05 g/L), vitamin K (10 ul/L), L-cysteine HCl (0.5 g/L), bile salts (0.5 g/L)) after the media was steamed for 20 min. Anaerobic conditions inside the vessels were maintained using a continuous stream of N_2_ (15 mL/min).

The pH within the vessels was maintained between 6.7 and 6.9 using a homeostatic pH controller (Electrolab, UK) connected to 0.5 M sodium hydroxide (NaOH) and hydrochloric acid (HCl). The temperature was kept at 37 ^0^C with circulating water on the outside of the vessel using a temperature-controlled water bath. For each donor there were nine vessels, seven of which contained the proteins of interest: pea (76% protein), egg (77%), whey (79%), milk (81%), fish meal (73%), and soya (80%), all of which were supplied by Food and Feed Innovations Ltd, and concentrated mycoprotein was donate by Quorn® Foods. Different quantities of the products were added to reach a comparable protein amount of 0.9 g in the vessels in order to simulate a 30-g bolus of protein that may be eaten in one sitting. The remaining two vessels were a negative control which did not contain additional protein, and a positive control (inulin (1.5 g); Beneo, Orafti® P95). Twenty-four hours prior to inoculation the vessels were maintained under anaerobic conditions, 37^0^C, and pH 6.7–6.9 to stabilise and reflect the environment of the descending colon. The vessels were then inoculated with 15 mL of faecal slurry (1:10, w/v) from a different volunteer each time and sample collection occurred at 0, 8, 24 and 48 h.

### Metabolomic and microbiota analyses

#### Preparation of samples for gas chromatography, and fluorescent in-situ -hybridisation coupled with flow cytometry (FISH *flow*).

At each time point 4 mL of sample were removed from each vessel. 1.5 mL of each sample were then placed in a 1.5-mL microcentrifuge tube and centrifuged at 13,000 *g* for 10 min and the supernatant was stored at − 20 °C for later GC analysis. A further 750 µL sample was removed for FISH*flow* analysis and placed in a microcentrifuge tube and spun at 13,000 *g* for 5 min. The supernatant was discarded, and the remaining pellet was aspirated with 375 µL PBS which was mixed with 1125 µL 4% paraformaldehyde solution. This solution was stored at 4 °C for 4 h before being washed twice with PBS. The pellet was then resuspended in 150 µL PBS and 150 µL ethanol before being stored at − 20 °C [40].

#### Ammonia production analysis assay

The procedure was followed as per the manufacturer’s instructions for the Ammonia Assay Kit (Sigma-Aldrich Co Ltd, AA0100). In brief, a 1 mM ammonia standard was prepared by mixing 10 µL of 20 mM NH_4_Cl with 190 µL H_2_O in a Falcon tube. The working reagent was prepared by combining the pre-prepared solutions of 90 µL ammonia assay buffer solution, 4 µL solution A and 4 µL solution B to each sample. The 1 mM standard was separated into a range of concentrations from 0 mM, 0.25 mM, 0.5 mM to 1 mM in the multiwell plate. A total of 10 µL of each sample to be assessed and 90 µL of the working reagent solution was placed into the remaining wells. The plate was mixed and incubated in the dark at room temperature for 15 min. Readings were taken at 360 nm (ex) and 450 nm (em) on a Tecan plate reader (Tecan, UK). The ammonia concentration for each sample was calculated using the calibration curve.

### Short-chain fatty acid and branched-chain fatty acid analysis using gas chromatography

The method used has been described previously [41]. Briefly, a mixture of 1 mL of sample, 50 µL internal standard (2-ethylbutyric acid), 0.5 mL concentrated HCL and 2 mL diethyl ether was vortexed at 1500 rpm for 1 min and centrifuged at 865 *g* for 20 min. The top diethyl ether layer was extracted and retained. The ether extract (400 µL) was mixed with 50 µL MTBSTFA (*N*-methyl-*N*-*tert*-butyldimethylsilyltrifluoroacetamide) and stored at room temperature for 48 h before analysis. An Agilent 7890B systems gas chromatograph (Hewlett Packard, UK) fitted with a flame ionisation detector and an HP-5ms column (30 × 0.25 mm, 0.25 mm film thickness (Agilent, UK)) was used for the analysis. The carrier gas used was helium at a flow rate of 1.9 mL/min and a head pressure of 139.8 kPa. The column temperature was programmed to increase from 63 °C to 190 °C at 5 °C per minute and held at 190 °C for 30 min, while the injector and detector temperatures were 275 °C. A split ratio of 100:1 was used. One µL of the samples were injected into the GC and HPChem software was used to record the peak areas of metabolites. The ratio of short-chain fatty acids (SCFAs) to branch-chain fatty acids (BCFAs) was determined by dividing the total concentration of SCFAs by BCFAs for each condition.

### Phenolic compounds analysed using headspace SPME with gas chromatography-mass spectroscopy

Volatile compound analysis was carried out by automated headspace solid-phase microextraction (SPME) coupled with gas chromatography-mass spectrometry (GC-MS), using an Agilent 110 PAL injection system mounted on an Agilent 7890 GC connected to a 5975 mass selective detector (MSD). The SPME fibre stationary phase was composed of 75 μm divinylbenzene/Carboxen™ on polydimethylsiloxane (Supelco, Bellefonte, PA). The samples (1 mL) were added and then equilibrated for 10 min at 35 °C before being extracted for 30 min. Samples were agitated at 500 rpm (5s on, 2s off) during equilibration and extraction. After extraction, the contents of the fibre were desorbed onto the front of a Zebron ZB5MS fused silica capillary column (30 m × 0.25 mm i.d., 1 μm film thickness; Phenomenex, Torrance, CA) in splitless mode, with the splitter opening after 0.75 min (100:1 split). The GC program and the fibre desorption step commenced at the same time. The GC oven was held at 60 °C before heating at 5 °C/min to 260 °C, where the temperature was maintained for 1 min. Helium was used as the carrier gas at a constant flow rate of 0.9 mL/min.

The mass spectrometer operated in electron impact mode with an electron energy of 70 eV, scanning from *m/z* 20 to *m/z* 280 at 1.9 scans/s. Compounds were identified using a library and their relative concentrations were determined by comparing peak areas with 1 µL of 10 mg/L internal standard (1,2-Dichlorobenzene).

### Microbiota analysis using fluorescence in situ hybridisation coupled with flow cytometry

Fluorescent in situ hybridisation coupled with flow cytometry (FISH*flow*) was used to quantify bacterial populations, with the method used by Rigottier-Gois et al. [42, 43] adapted for the Accuri C6 flow cytometer. The frozen fixed samples (-20^0^C) were thawed and vortexed for 10 s. Then 75 µL of each sample was mixed with 500 µL of PBS in a 1.5 mL Eppendorf tube, followed by centrifugation at 11,337 *g* for 3 min. The resulting supernatant was discarded and a mixture of 100 µL of Tris-EDTA buffer (pH 8.0) with lysozyme (1 mg/mL) was added to the pellet. After stirring, the samples were incubated in the dark for 10 min before being vortexed and centrifuged again at 11,337 *g* for 3 min. The supernatants were removed, and the pellets were resuspended in 500 µL of PBS, vortexed, and centrifuged (11,337 *g* for 3 min). Subsequently, the pellets were resuspended in 150 µL of hybridisation buffer (0.9 M NaCl, 0.2 M Tris-HCl (pH 8.0), 0.01% sodium dodecyl sulphate, 30% formamide), vortexed, and centrifuged (11,337 *g* for 3 min). Supernatants were removed and pellets were resuspended in 1 mL of hybridisation buffer. Oligonucleotide probe solution (50 ng/mL, 4 µL) (Table 1) was added to 50 µL of each sample in 1.5 mL Eppendorf tubes, vortexed, and incubated overnight at 36^0^C, which was previously determined to be the optimal temperature for the probes [40]. On the following day, each sample was centrifuged (11,337 *g* for 3 min) and washed with 125 µL of hybridisation buffer, vortexed, and centrifuged (11,337 *g* for 3 min). Supernatants were removed and pellets were resuspended in 175 µL of washing buffer solution (0.064 M NaCL, 0.02 M Tris/HCL (pH 8.0), 0.5 EDTA M (pH 8.0), 0.01% sodium dodecyl sulfate). After vortexing and incubating in the dark at 35^0^C to eliminate non-specific probe binding, the samples were vortexed and centrifuged again and the supernatants were discarded. Pellets were resuspended in 300 µL of PBS, vortexed and stored in the dark at 4^0^C before being analysed using the BD Accuri™ C6 flow cytometer (BD, Brussels) at excitation wavelengths of 488 nm and 640 nm. Data analysis was performed using the Accuri CFlow Sampler Software.

**Table 1:** Oligonucleotide probe sequences

Probe name	Target Species	Sequence
Non Eub	Control Probe	ACTCCTACGGGAGGCAGC [44]
Eub338I+	Majority of bacteria	GCTGCCTCCCGTAGGAGT
Eub338II+	*Planctomycetales*	GCAGCCACCCGTAGGTGT [45]
EUB338III+	*Verrucomicrobiales*	GCTGCCACCCGTAGGTGT [45]
Bif164	*Bifidobacterium* spp.	CATCCGGCATTACCACCC [46]
Lab158	*Lactobacillus* and *Enterococcus*	GGTATTAGCAYCTGTTTCCA [47]
Bac303	Bacteroidaceae, prevotellaceae and some porphyromonadaceae	CCAATGTGGGGGACCTT [48]
Erec482	Most *Clostridium coccoides-Eubacterium rectale* group	GCTTCTTAGTCARGTACCG [49]
Rrec584	*Roseburia* genus	TCAGACTTGCCGYACCGC [50]
Ato291	*Atopobium* cluster	GGTCGGTCTCTCAACCC [51]
Prop853	*Clostridium* cluster IX	ATTGCGTTAACTCCGGCAC [50]
Fprau655	*Faecalibacterium prausnitzii*	CGCCTACCTCTGCACTAC [52]
DSV687	*Desulfovibrio* genus	TACGGATTTCACTCCT [53]
Chis150	*Clostridium histolyticum*	TTATGCGGTATTAATCTYCCTTT [49]

### Statistical analyses

General linear modelling was conducted using IBM SPSS statistics version 27.0.1 (IBM, Chicago, IL, USA) on the individual metabolite and microbial functional groups present, with ‘treatment’ or ’protein type’ or ‘additional protein’ as factors along with ‘sex’ and ‘time’. Multiple test corrections were completed using least significant difference (LSD).

## Results

### Fermentation of dietary proteins derived from different sources resulted in significant shifts in microbiota composition

**Fig. 1:**
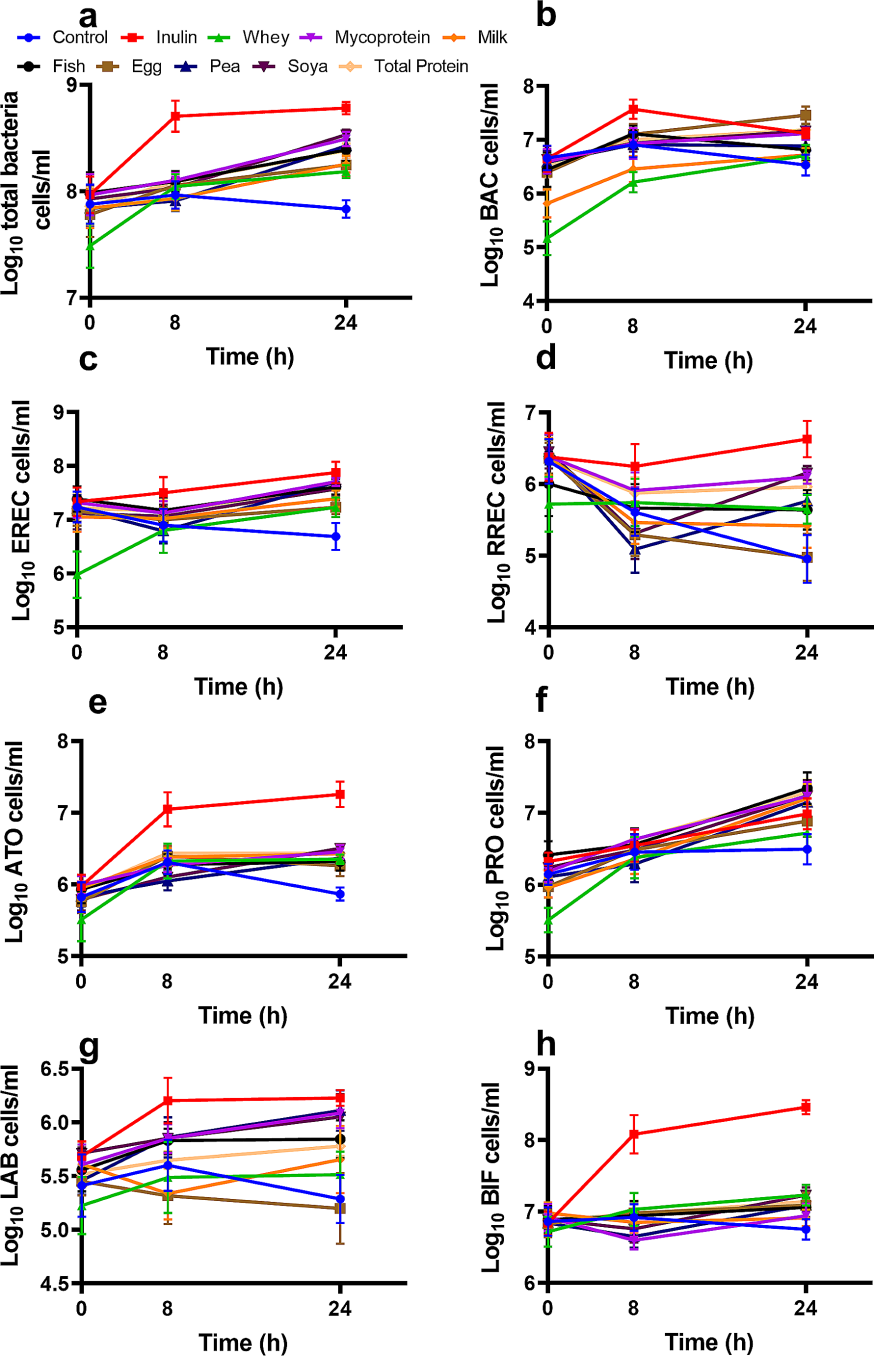
Effects of fermentation of proteins extracted from different sources (pea, whey, egg, milk, fish meal, soy and mycoprotein), inulin and negative control (no substrate) on bacterial populations from human faeces following inoculation of anaerobic, pH controlled, batch culture systems and quantification by FISH*flow* at 0, 8 and 24 h. Averages of the results of all protein samples for each individual volunteer were calculated to generate the “total protein” values. Bacterial groups quantified were total prokaryotes (EUB, **a**), *Bacteroidaceae* and *Prevotellaceae* (BAC,**b**), *Clostridium coccoides-Eubacterium rectale* group (EREC, **c**), *Roseburia* cluster (RREC, **d**), *Atopobium* Cluster (ATO, **e**), *Clostridium* Cluster IX (PRO, **f**), *Lactobacillus* spp. (LAB, **g**), *Bifidobacterium* spp. (BIF, **h**). Data shown are means of 10 independent experiments (*n = 5* males and 5 females) ± SEM. General liner modelling was used and LSD multiple testing correction was applied to generate the table of significances for the model. Significances of *p* < 0.05 are highlighted. Significance of individual differences can be viewed in Supplementary Table 1

**Fig. 2:**
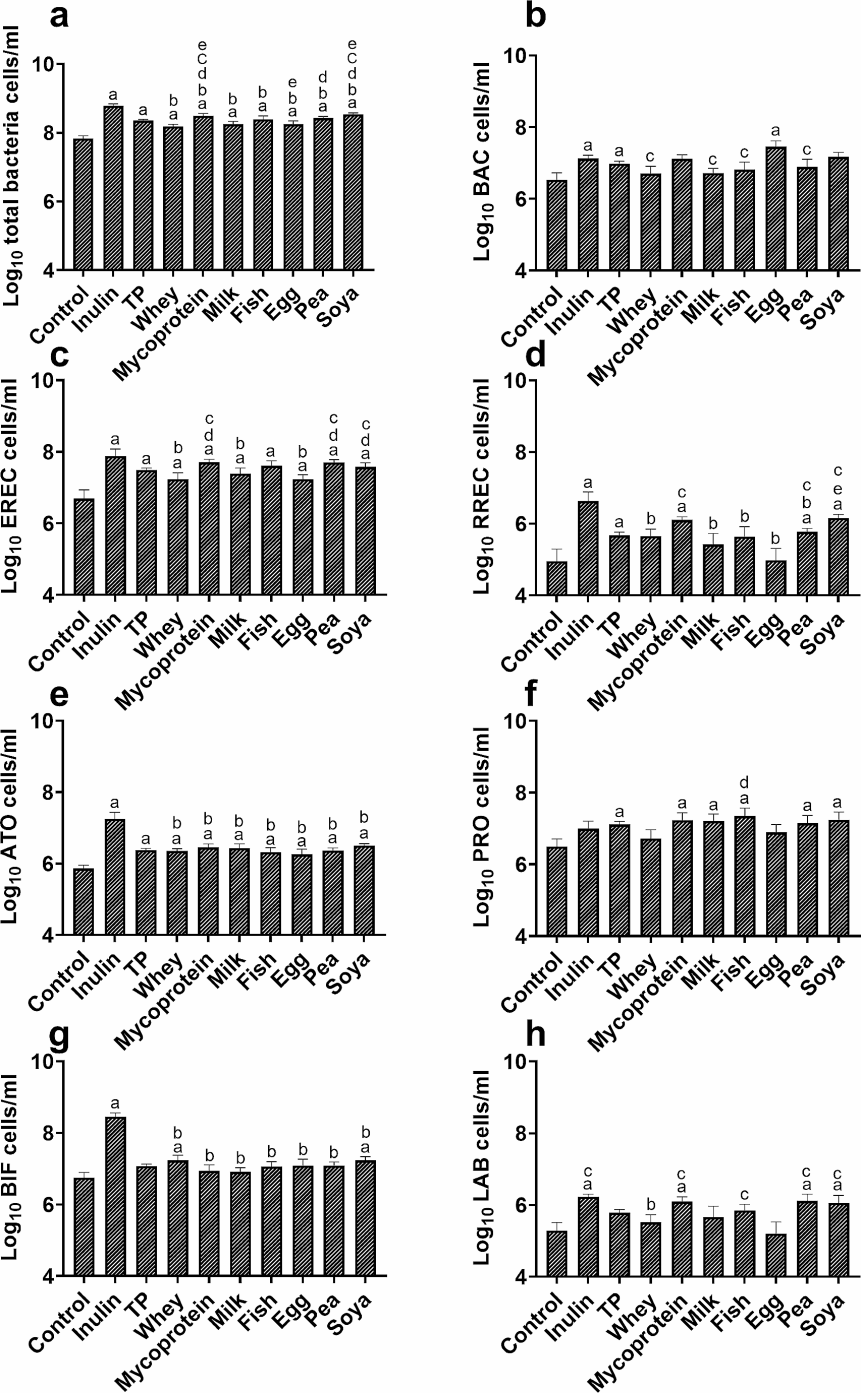
Proteins isolated from different sources (pea, whey, milk, fish meal, egg, soy and mycoprotein), and inulin, were fermentable substrates utilised by faecal bacteria in anaerobic batch culture systems, along with a negative control (no substrate). These results are from the 24 h timepont in Fig. 1. The results of all the proteins from each individual participants were averaged and labelled as total protein (TP). This was used to compare the effect of overall protein fermentation on bacterial populations against the negative control. FISH*flow* was used to quantify the following bacterial populations: total prokaryotes (EUB, **a**), *Bacteroidacea* and *Prevotellaceae* (BAC, **b**), *Clostridium coccoides-Eubacterium rectale* group (EREC, **c**), *Roseburia* cluster (RREC, **d**), *Atopobium* Cluster (ATO, **e**), *Clostridium Cluster* IX (PRO, **f**), *Bifidobacterium* spp. (BIF, **g**), *Lactobacillus* spp. (LAB, **h**). Letters denote a significant difference between conditions: a = significantly different to control, b = significantly different to inulin, c = significantly different to egg, d = significantly different to whey, e = significantly different to milk. Data shown are means of 10 independent experiments (5 males; 5 females) ± SEM

The effects of increased protein availability on the composition of the gut microbiota were explored using in vitro fermentation systems and human faecal inoculate. We observed significant main effects of ‘treatment’ in the overall model for all bacteria and functional groups explored (all bacteria (EUB), *p* < 0.001; *Bifidobacterium* (BIF), *p* < 0.001; *Lactobacillus-Enterococcus* group (LAB), *p* = 0.035; *Bacteroides* spp (BAC), *p* < 0.001; *Clostridium coccoides* group (EREC), *p* < 0.001; *Roseburia* spp. *(*RREC), *p* < 0.001; *Atopobium* cluster (ATO), *p* < 0.001; *Clostridium* cluster IX (PRO), *p* < 0.001). Total protein (TP) effects were obtained by averaging the values from individual proteins and not by including all proteins simultaneously in gut model vessels. Specifically, at the 8 h timepoint, the effects of the different protein sources on the microbial community were not apparent (Fig. 1a-h), However, after 24 h the fermentation of total protein led to increased numbers of total bacteria (EUB) (Fig. 2a, *p* < 0.001), BAC (Fig. 2b, *p* = 0.005), EREC (Fig. 2c, *p* = 0.004), RREC (Fig. 2d, *p* = 0.009), ATO (Fig. 2e, *p* < 0.001), and (PRO (Fig. 2f, *p* = 0.009) compared to equivalent negative controls, in which additional proteins were absent. No significant differences were observed in abundances of BIF (Fig. 2g) genus or *Lactobacillus-Enterococcus* group (Fig. 2h), *Desulphovibrionaceae* or *Clostridium histolyticum* group (data not shown), nor at other timepoints for TP. No analysis of microbiota composition was conducted at 48 h as bacterial fermentation in the colon likely would not last 48 h in vivo.

There were differences in the expansion of bacterial groups following fermentation of proteins from differential sources at 8 h (Fig. 1a-h). At 8 h, both mycoprotein (*p* = 0.035) and pea protein (*p* = 0.038) fermentation resulted in significantly elevated levels of EREC in comparison to whey and egg proteins. Whey protein fermentation also resulted in significantly fewer BAC than mycoprotein (*p* = 0.021), fish (*p* < 0.01), egg (*p* < 0.01), pea (*p* = 0.025) and soya (*p* = 0.02) proteins, while milk protein fermentation resulted in significantly lower levels of BAC than fish (*p* = 0.035) and egg (*p* = 0.038) all at 8 h. By 24 h, soy protein fermentation resulted in significant increases in all bacterial groups (*p* < 0.05, Fig. 2a-h) except for BAC (Fig. 2b), whereas egg protein was only able to stimulate increased growth of BAC (*p* < 0.001), EREC (*p* = 0.018) and ATO (*p* = 0.019) compared to negative controls, which contained no additional protein. Taken together, these findings demonstrate that the source of protein is a key factor when considering the effects of protein on microbial populations. The *p*-values generated following statistical comparisons between all bacterial functional groups and all protein sources at each timepoint are available in Supplementary Table 1.

### Expansion of specific bacterial functional groups in response to protein fermentation significantly differed between the sexes

**Fig. 3:**
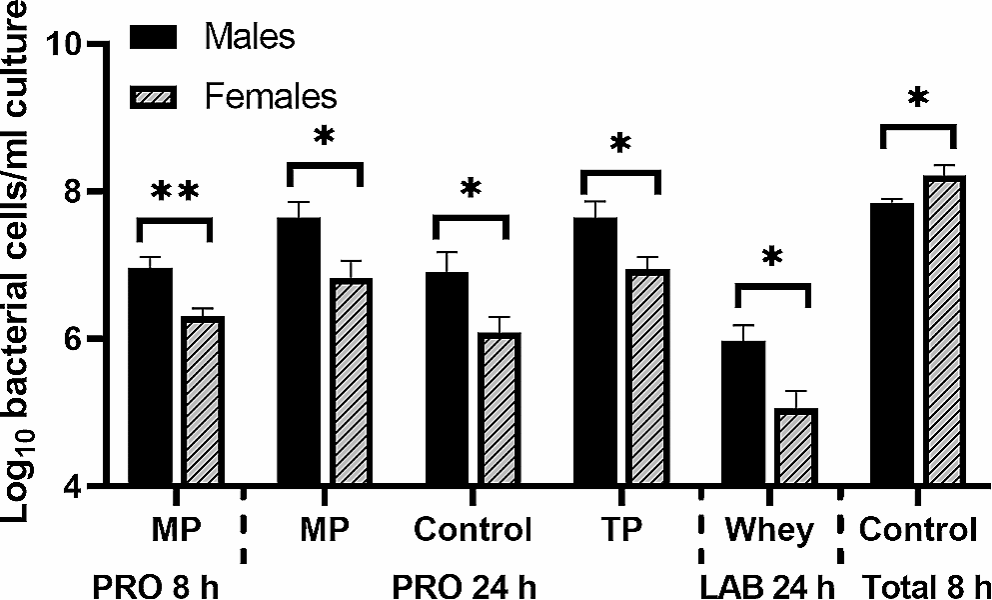
Sex differences in the effect of fermentation of proteins isolated from different sources and negative control (no substrate) in the abundance of bacterial groups *Clostridium* cluster IX (**PRO**) at 8 and 24 h, *Lactobacillus-Enterococcus* group (**LAB**) at 24 h, and total bacteria at 8 h (**d**)) from healthy human faeces inoculated into anaerobic, pH controlled, batch culture systems. **MP** = Mycoprotein. Samples from 0, 8 and 24 h were quantified by FISH*flow*. Values are means from 5 males and 5 females ± SEM. * = *p* < 0.05, ** = *p* < 0.01

Faecal samples from 5 healthy males and 5 healthy females were used to inoculate in vitro fermentation systems. When all conditions were included in the model, ‘sex’ was found to have significant main effects (*p* < 0.05) for each group of bacteria quantified (other than BAC) at 24 h. Here, total protein (TP) fermentation produced significantly increased numbers of PRO by males compared to females (*p* = 0.019) (Fig. 3). Sex differences were also found following bacterial fermentation of individual proteins. PRO was more abundant in males than females at 8 h in response to mycoprotein fermentation (*p* = 0.006) and for mycoprotein (*p* < 0.01) and control (*p* = 0.03) at 24 h. We also observed a trend for the numbers of PRO being greater in the male stool inoculated fermenters compared to females following soya protein fermentation at 24 h, but this did not reach significance (*p* = 0.058). Additionally, the male microbiota resulted in higher numbers of LAB at 24 h than females after fermenting whey protein (*p* = 0.019), whilst the female microbiota had significantly more total bacteria than males following soya protein fermentation at 8 h (*p* = 0.038). No sex differences in microbial populations were found at baseline. Here we show that the effects of different types of protein fermentation on the gut microbiota were dependent on the sex of the donor. To view all other bacterial counts for males and females view Table 3 in the supplementary material.

### Bacterial fermentation of dietary proteins influenced the production of both beneficial and potentially detrimental microbial-derived metabolites

**Fig. 4:**
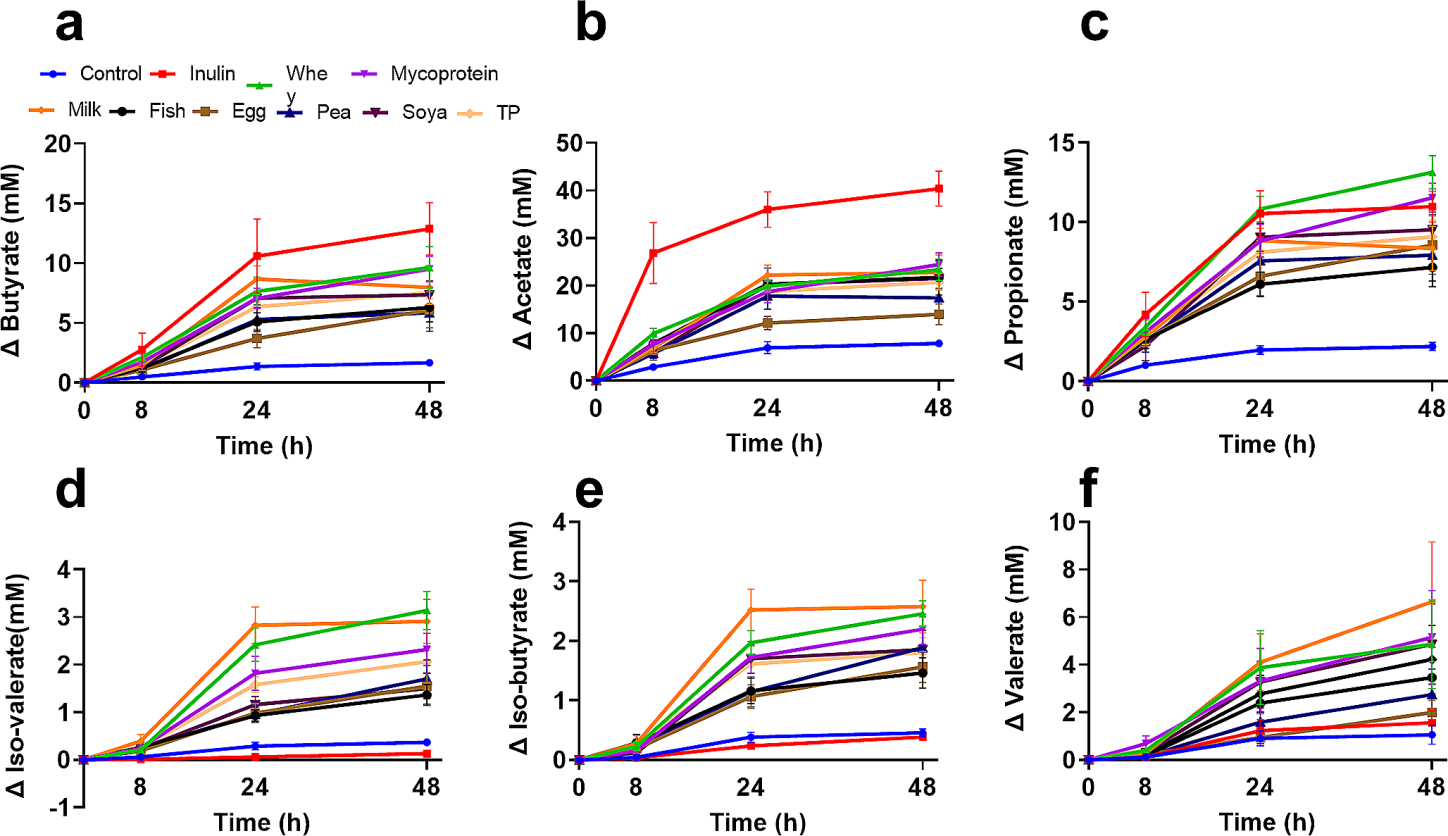
Effect of faecal bacteria fermentation of different protein substrates (pea, whey, milk, fish meal, egg, soy and mycoprotein) and inulin on the concentrations of short-chain fatty acids butyrate (**a**), acetate (**b**) and propionate (**c**), and on branched-chain fatty acids iso-valerate (**d**), iso-butyrate (**e**), valerate (**f**) quantified by GC/MS at 0 (baseline), 8, 24 and 48 h. The means of all the protein substrates used were averaged for each individual and labelled as total protein (TP). Human faecal inoculums from 10 healthy donors (5 males; 5 females) were used to inoculate anaerobic, pH and temperature-controlled batch culture systems. Error Bars = SEM General linear modelling using ‘sex’, ‘treatment’ and ‘time’ as factors, with LSD multiple testing correction, was used to analyse the model. Significances of *p* < 0.05 are highlighted. For individual significant differences, see supplementary Table 2

**Fig. 5:**
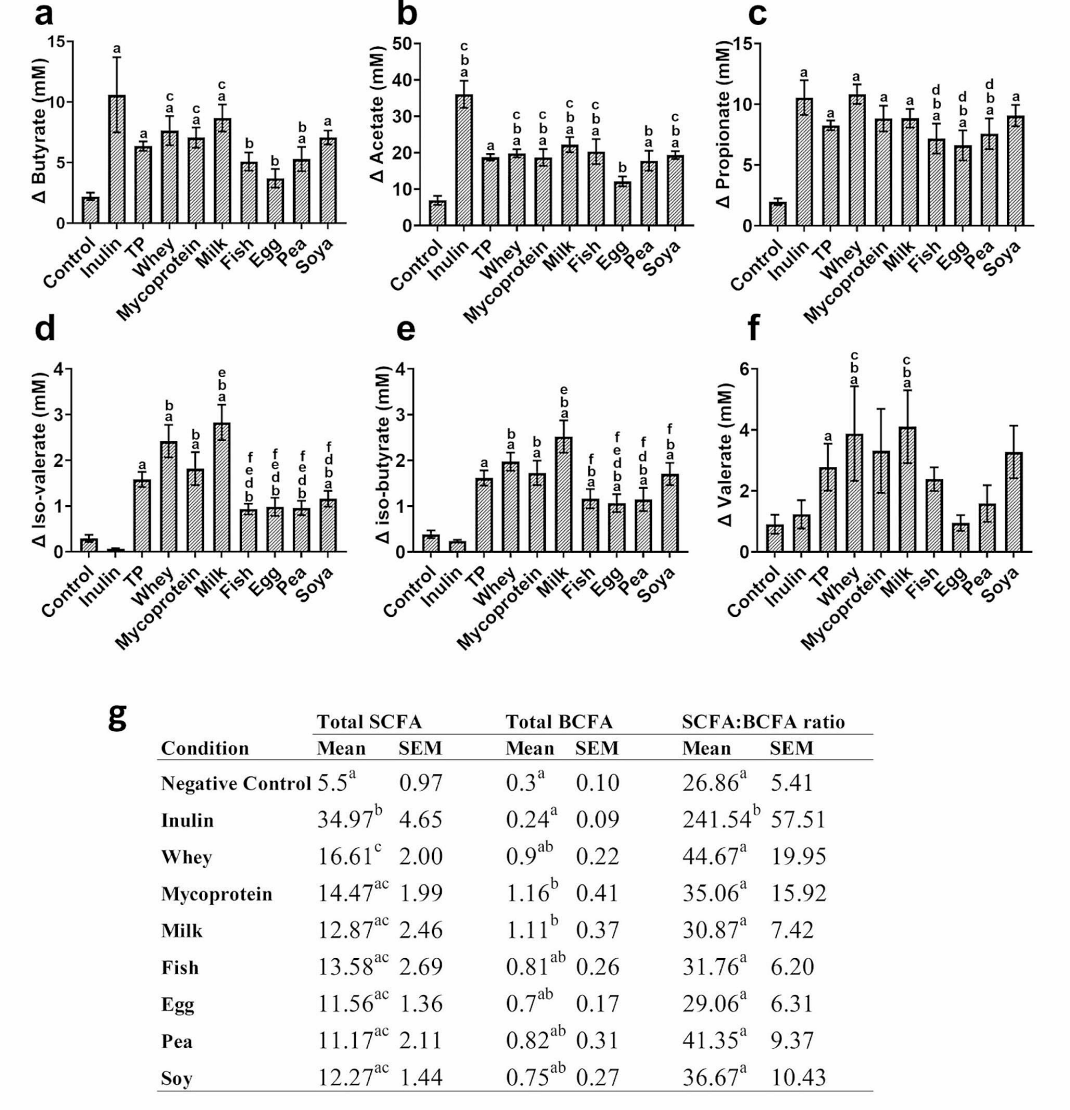
Effects of bacterial fermentation of different dietary proteins, inulin and negative control (no substrate) by human faecal microbiota on the concentration of phenol (**a**), p-cresol (**b**), indole (**c**) and ammonia (**d**) at 24 h in *in-vitro*, pH controlled, anaerobic, batch culture systems. Phenol, indole and p-cresol were quantified using GC/MS, ammonia was analysed using ammonia assay. The average was determined from the results of all proteins and is labelled as total protein (TP) and was compared against the control. Data shown are means (**a**, **b** and **c**) and mean change from baseline (**d**) of 10 independent experiments with different donors (*n* = 5 males and females). Error bars = SEM. Letters denote significant differences between conditions: a = significantly different to control, b = significantly different to inulin, c = significantly different to egg, d = significantly different to whey, e = significantly different to milk

Gas chromatography coupled with mass spectrometry was used to quantify microbial-derived end-product metabolites, including short-chain fatty acids (SCFAs, Fig. 4a-c), branched-chain fatty acids (BCFAs) (Fig. 4d-f) and phenolic compounds (Fig. 5) after 0, 8, 24 and 48 h of fermentation with each of the substrates. Statistical modelling showed that all SCFAs and BCFAs had significant main effects of ‘treatment’ (*p* ≤ 0.001). With regards to beneficial SCFAs, increases in butyrate production following fermentation of total protein (TP) was significantly greater than negative controls at 8 (*p* = 0.037), 24 (*p* < 0.01) and 48 (*p* < 0.01) h (Fig. 4a). Acetate (Fig. 4b**)** and propionate (Fig. 4c) production in response to total protein (TP) fermentation compared to negative controls was significantly increased by 24 and 48 h (*p* < 0.01 for both). Concentrations of the branched-chain fatty acids (BCFAs) iso-valerate (Fig. 4d, *p* < 0.01) and iso-butyrate (Fig. 4e, *p* < 0.011) were significantly higher following total protein fermentation at all time points compared to negative controls, whereas valerate (Fig. 4f, *p* = 0.037) only increased at 24 h. No differences between males and females were found at 0 h. Correlation analyses of the microbiota composition and metabolite production can be found in supplementary Table 2.

**Fig. 6:**
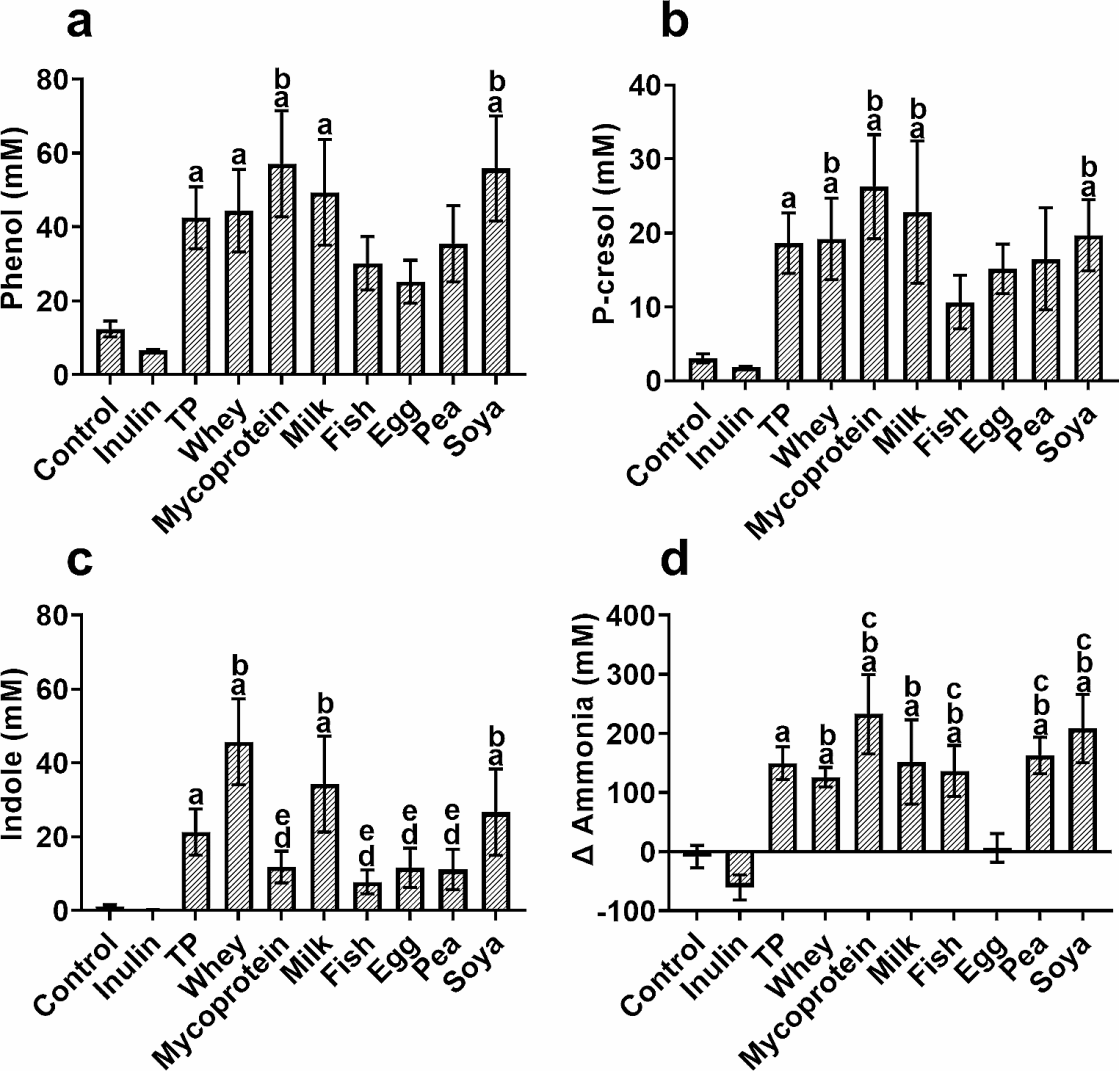
Anaerobic faecal batch culture systems designed to reflect conditions in the human colon (pH controlled at 6.7–6.9, temperature controlled at 37^o^C) were used to explore the production of microbial-derived metabolites following fermentation of different dietary proteins (pea, whey, milk, fish meal, egg, soy and mycoprotein) and inulin compared to a negative control (no substrate). The production of short chain fatty acids butyrate (**a**), acetate (**b**) and propionate (**c**), and branched chain fatty acids iso-valerate (**d**), iso-butyrate (**e**) and valerate (**f**) were quantified using GC/MS. Average was determined from the results of all proteins from each individual and are labelled ‘total protein’ (TP) and were compared against the negative control. Figure 5g presents the aggregated concentrations of SCFAs and BCFAs quantified for each condition at 24 h, alongside the corresponding ratio of SCFAs to BCFAs. Letters denote significant difference between conditions. Data presented are the mean changes from baseline of 10 independent experiments with different donors (*n* = 5 males and 5 females) at the 24 h time point from Fig. 4 ± SEM. Letters denote a significant difference between conditions: a = significantly different to the negative control, b = significantly different to inulin, c = significantly different to egg, d = significantly different to whey, e = significantly different to mycoprotein, f = significantly different to milk

Production of SCFAs by bacteria as a result of protein fermentation was highly dependent on the protein source. All individual protein sources led to significantly more butyrate production (*p* < 0.05) than the negative controls, except for fish and egg proteins (Fig. 6a). In addition, egg protein fermentation was associated with lower butyrate concentrations compared to whey (*p* = 0.045) and milk (*p* = 0.012), and less acetate (Fig. 6b) than whey (*p* = 0.031), milk (*p* < 0.01), fish (*p* = 0.023), and soya (*p* = 0.023), and also less propionate (Fig. 6c) than whey protein fermentation (*p* < 0.01) at 24 h. No significant differences were observed between protein sources in terms of change in SCFA concentration at 8 h. In comparison to the negative controls, milk protein fermentation resulted in higher levels of BCFAs including iso-valerate (*p* = 0.012) at 8 h (Fig. 4d), while milk, whey and mycoprotein (*p* < 0.01 for all) fermentations resulted in increased concentrations of iso-butyrate (Fig. 4e) at 8 h. In addition, fermentation of mycoprotein led to elevated levels of valerate (*p* = 0.01, Fig. 4f) compared to fish (*p* = 0.024), pea (*p* = 0.017) and egg (*p* = 0.037), all at 8 h. By 24 h, is—valerate production (Fig. 6d) was significantly greater following fermentation of whey (*p* < 0.001), mycoprotein (*p* = 0.01) and milk (*p* < 0.001) compared to fish protein. Fermentation of whey (*p* < 0.01), mycoprotein (*p* = 0.04) and milk (*p* < 0.001) also resulted in significantly higher concentrations of iso-butyrate (Fig. 6e) than egg protein. Similarly, the increase in valerate concentrations at 24 h was significantly greater when both whey (*p* = 0.025) and milk (*p* = 0.016) proteins were utilised by the faecal bacteria in comparison to egg protein. After 24 h, inulin fermentation led to a significantly greater SCFA: BCFA ratio when compared to negative control and all protein conditions. However, the protein conditions themselves did not show any differences in the SCFA: BCFA ratio, as illustrated in Fig. 6g. All values for significant differences between proteins for SCFA and BCFA concentrations can be found in Supplementary Table 2.

### The origin of protein and sex of the donor determine the extent of phenolic compound production following bacterial fermentation

Microbial-derived phenolic compounds have been linked with reduced intestinal barrier function in vitro, and we observed significant main effects of ‘treatment’ for all phenolic compounds assessed (*p* < 0.019). Increased total protein availability (TP) was associated with increases in the production of phenol (*p* < 0.01, Fig. 5a), *p*-cresol *(p* < 0.01, Fig. 5b), indole (*p* = 0.018, Fig. 5c) and ammonia *(p* < 0.01, Fig. 5d) at 24 h compared to negative controls. There were also significant interactions between levels of phenolic compound production and the specific type of protein being fermented by the gut-derived bacteria. Here, fermentation of mycoprotein (*p* = 0.03) and soy (*p* = 0.037) both resulted in significantly higher concentrations of phenol than egg-derived protein (Fig. 5a). Concentrations of *p*-cresol were also significantly higher as a result of mycoprotein fermentation in comparison to fish (*p* = 0.047), while whey protein fermentation resulted in significantly higher concentrations of indole than mycoprotein (*p* = 0.001), fish (*p* = 0.001), egg (*p* = 0.002) and pea (*p* = 0.02) protein fermentation (Fig. 5c). In response to both soy (*p* = 0.001) and mycoprotein (*p* < 0.001) fermentation, the gut derived bacteria increased ammonia production by around 200 mM whereas fermentation of egg protein did not result in detectable increases in ammonia production (Fig. 5d). All *p*-values denoting significances in phenolic compound production in response to individual protein fermentation can be found in Supplementary Table 2.

**Fig. 7:**
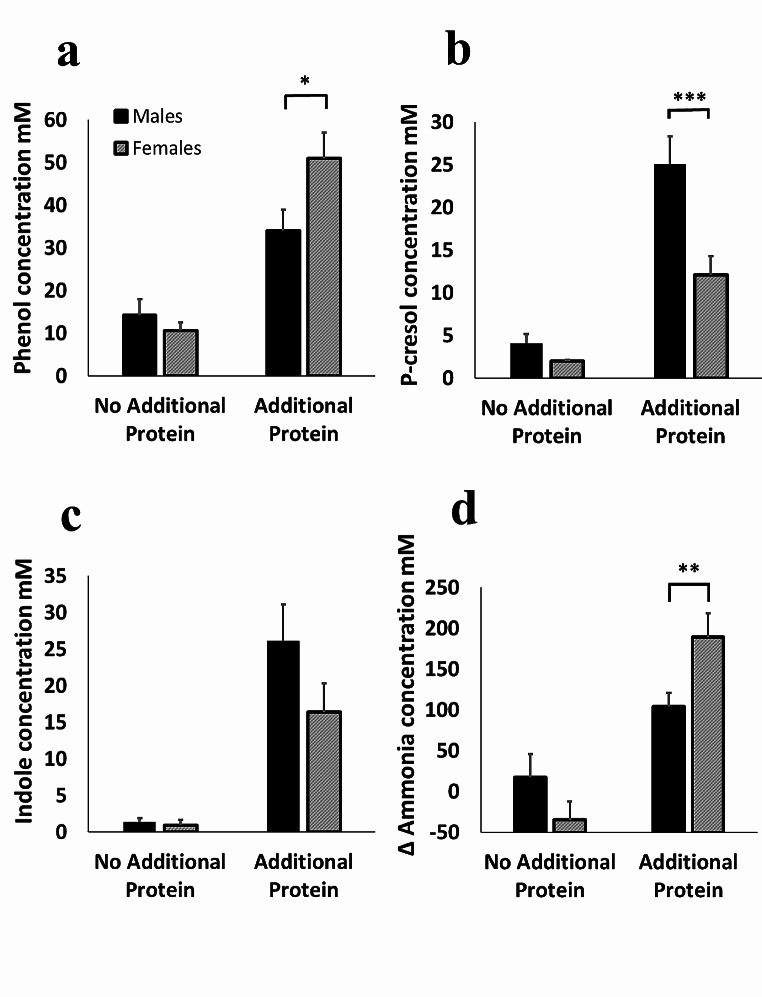
The effect of increased dietary protein availability (pea, whey, milk, fish meal, egg, soy and mycoprotein) on the production of metabolites associated with reductions in gut barrier function (phenol (**a**); p-cresol (**b**); indole (**c**); ammonia (**d**)) was assessed using an *in-vitro* gut model system (pH controlled at 6.7–6.9, temperature controlled at 37 ^o^C). The values for ‘additional protein’ were calculated as an average of the results from the use of each of the proteins fermented individually and compared to the negative control (no substrate). Data presented are from 10 independent experiments with different donors (*n* = 5 males and 5 females) at the 24 h time point. Error bars ± SEM, * *p* < 0.05, ** *p* < 0.01, *** *p* < 0.001

**Fig. 8:**
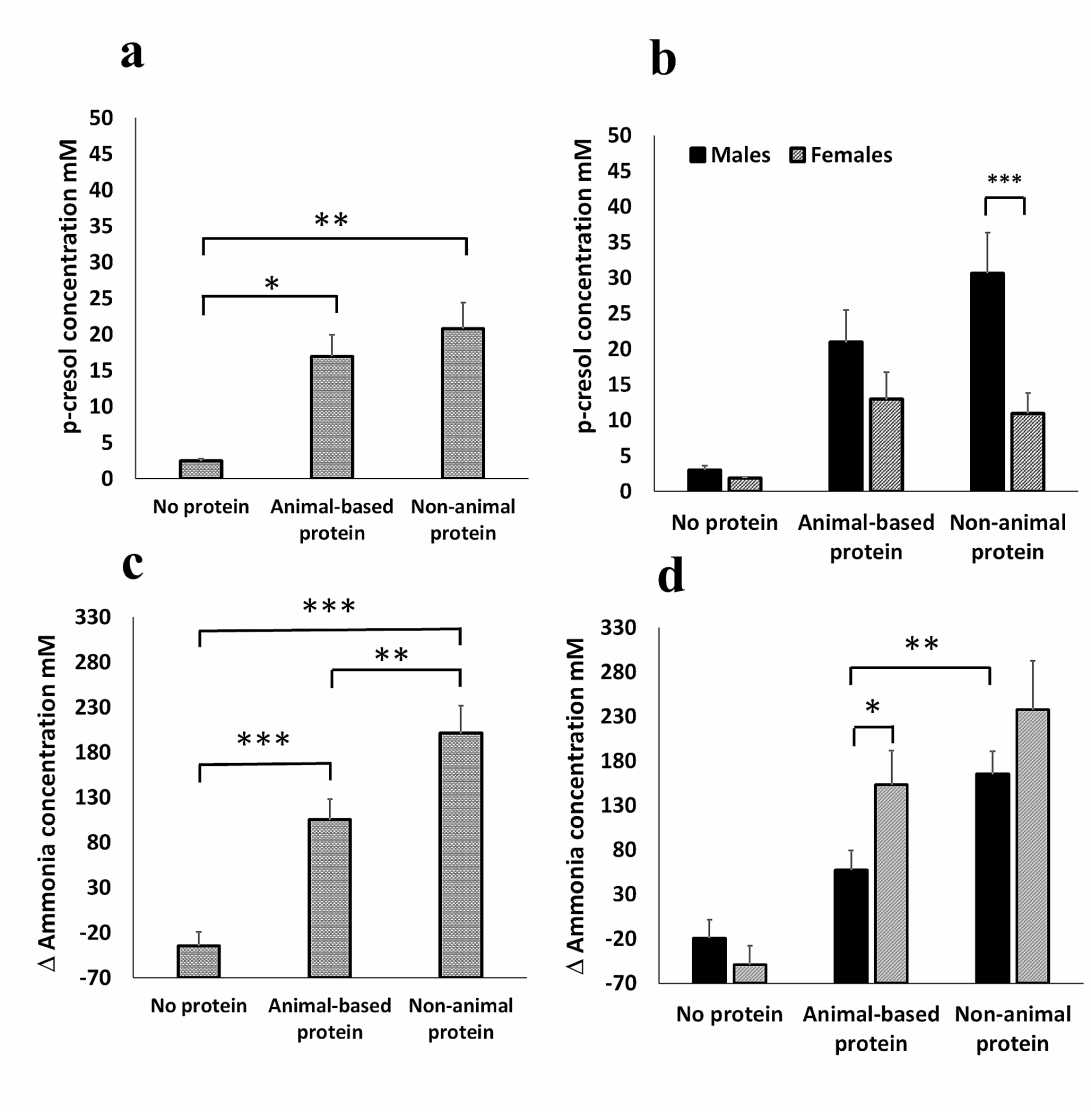
Dietary protein isolated from animal (milk, whey, fish meal and egg) and non-animal- based proteins (soy, pea and mycoprotein) were utilised as fermented energy sourced by faecal bacteria in anaerobic gut modelling systems (pH controlled at 6.7–6.9, temperature controlled at 37 ^o^C). The production of metabolites potentially detrimental to gut barrier function was analysed after 24 h of fermentation. Each protein was fermented individually and the results from non-animal and animal-based protein were determined from the average of the proteins within the respective categories. These categories were then compared against the negative control (no protein). Data presented are from 10 independent experiments, from *n* = 5 males and females which are shown together (**a** & **c**) or separately (**b** & **d**). Error bars ± SEM, * *p* < 0.05, ** *p* < 0.01, *** *p* < 0.001

The extent of the effect of protein fermentation on phenolic compound production at 24 h was highly dependent on the sex of the donor. Gut bacteria from females produced higher concentrations of phenol (*p* = 0.032, Fig. 7a) and ammonia (*p* = 0.012, Fig. 7d), while microbes from males produced higher concentrations of *p*-cresol (*p* = 0.001, Fig. 7b). Sexual dimorphism in phenolic compound production was a direct consequence of protein fermentation, as there were no significant sex differences in the absence of additional proteins (Fig. 7a-d). In addition, production of indole did not appear to be sex-dependent (Fig. 7c). There were no significant differences between fermentation of animal (whey, fish, milk and egg) and non-animal (soy, pea and mycoprotein) proteins in the productions in *p*-cresol (Fig. 8a); however, ammonia production was increased following fermentation of non-animal protein than animal proteins (*p* = 0.003) (Fig. 8c). Furthermore, male and female microbiotas produced similar concentrations of *p*-cresol following fermentation of animal proteins, but male-derived microbiotas produced higher concentrations of *p*-cresol (*p* = 0.004) than female-derived microbiotas in response to non-animal proteins, whereas microbiotas from females produced more ammonia (*p* = 0.035) following fermentation of animal proteins compared to that from males (Fig. 8d).

### Microbial-derived metabolite production in response to specific protein fermentation occurred in a sex-dependent manner

**Fig. 9:**
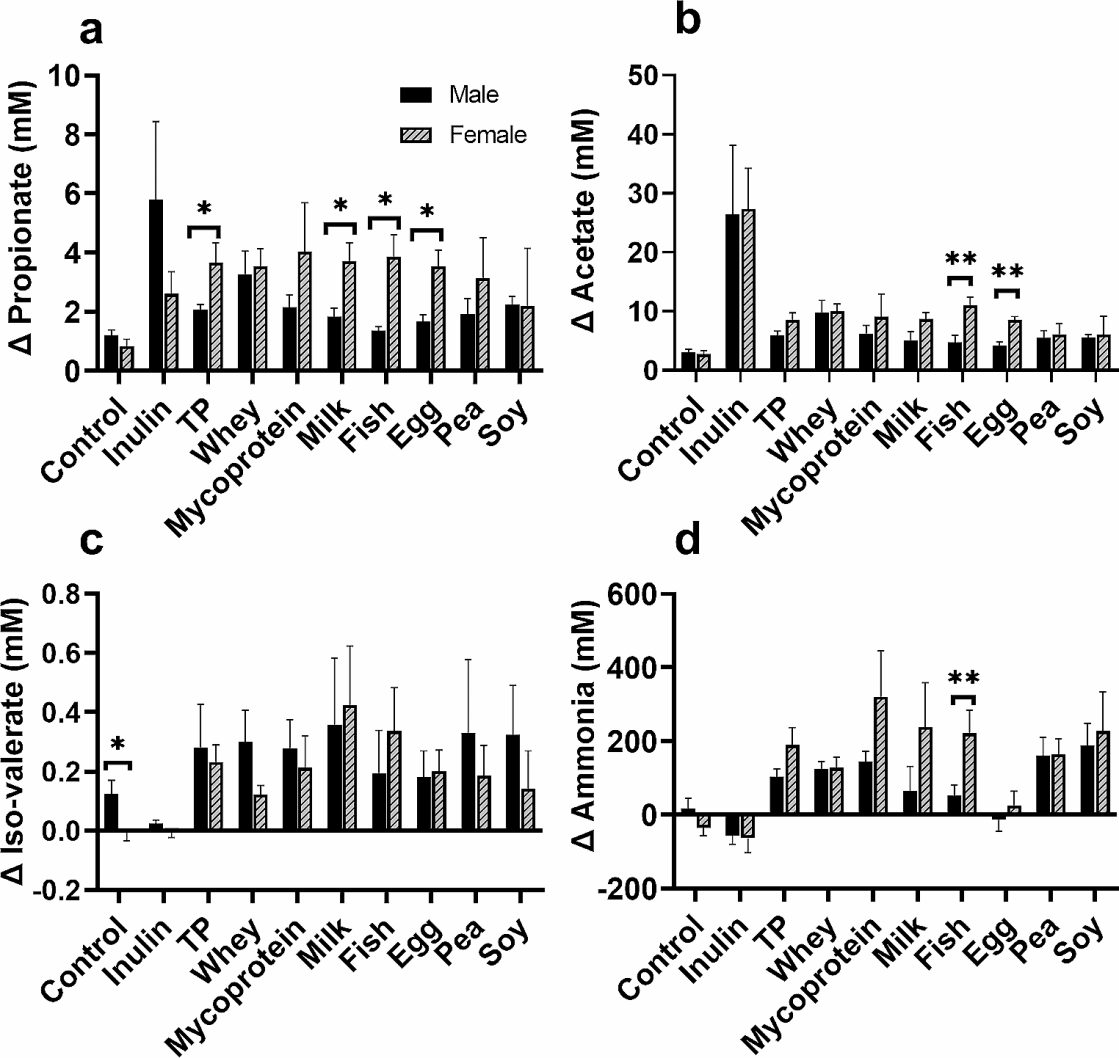
Sex differences in the production of metabolites (propionate at 8 h (**a**), acetate at 8 h (**b**), iso-valerate at 8 h (**c**) and ammonia at 24 h (**d**) following fermentation of different dietary proteins, and control (no substrate) by human faecal microbiota in batch culture systems. Acetate and propionate levels were quantified using GC/MS, and ammonia was quantified with an ELISA. Values are means from 10 independent experiments (*n* = 5 males and females) ± SEM. * = *p* < 0.05, ** = *p* < 0.01

Finally, we identified sex differences in the production of microbial-derived metabolites following fermentation of specific proteins. At 8 h, the female-associated microbiota produced significantly more propionate following fermentation of milk (*p* = 0.026), fish (*p* = 0.011) and egg (*p* = 0.013) proteins than the male-associated microbiota (Fig. 9a), and significantly higher concentrations of acetate following fish (*p* < 0.01) and egg (*p* = 0.001) utilisation (Fig. 9b), also at 8 h. Male-derived microbiotas produced significantly more iso-valerate (*p* = 0.001) in the absence of any protein fermentation than female microbiotas did at 8 h (Fig. 9c), but there were no further sex-dependent differences in iso-valerate production following fermentation of specific proteins. However, female-derived microbiotas produced higher concentrations of ammonia following fermentation of fish-derived proteins (*p* = 0.02). No significant differences between males and females were found for the other metabolites measured or for any of the metabolites at 0 h.

## Discussion

Despite recent recommendations to increase protein consumption in adults [1], the effects of high protein diets on the composition and metabolic output of the gut microbiota, and potential to disrupt intestinal barrier function, remain largely uncharacterised. Using in vitro systems, we show that bacterial fermentation of protein caused shifts in microbiota composition towards more proteolytic phenotypes including *Bacteroides* and *Clostridium* genera. However, the significance of these increases was highly dependent on the source of the protein. In addition, fermentation of proteins from different sources resulted in differential concentrations of potentially gut barrier-disrupting bacterial-derived metabolites, including phenol, *p*-cresol, indole and ammonia. We also demonstrate that the responses of the microbiota to increased protein availability weresex-specific, with significant interactions occurring between the proteins originating from different sources and the sex of the donors. This could have important implications with regards to providing appropriate dietary advice and guidelines to different populations to reduce risks of developing chronic diseases of the cardiovascular system and liver.

As is consistent across all reductionist biological in vitro models, gut fermentation systems do not fully reflect physiological conditions within the human colon due to the absence of, for example, hormones and immune-associated molecules and cells. In addition, proteins entering the colon would normally have undergone some degree of physiological modifications by digestive enzymes. However, these fermentation models do provide a supportive environments for the microbiota, with limited additional nutrients, to permit evaluation of the direct effect of the test substrate, in this case protein, on bacterial populations and microbial metabolic activity. Indeed, since the models lack absorptive capacity, microbial derived metabolites accumulate in the model and production can be quantified in response to protein availability, which would be challenging in animal or human studies. Furthermore, we have previously demonstrated that findings derived from our gut model systems often reflect findings from subsequent human trials [54–56].

Increased protein availability was linked with significant shifts in the composition of the gut microbiota, with 7 out of the 11 functional groups quantified, differing significantly from the controls. Two of the most notable bacterial functional groups that were less prevalent under increased protein availability were *Bifidobacterium* and *Lactobacillus* spp genera. These groups include the most commonly used bacteria for probiotic supplementation, due to the large body of evidence that supports their positive effects on health [57–59], including reductions in intestinal permeability [60, 61]. In our trial, four phenolic metabolites (phenol, *p*-cresol, indole and ammonia), which have been shown to reduce barrier function in vitro [12], were significantly elevated under high protein conditions compared to the low protein conditions of the controls. These results are in accordance with a previously published study which also used *in-vitro* gut model systems [62]. However, most studies exploring the effects of high protein availability on microbiota composition and/or metabolic activity used hydrolysed protein in order to obtain high purity (∼ 95%), for example, casein hydrolysates [9, 62]. These ultra-concentrated sources of protein, limit levels of the non-protein substrates usually associated with these proteins when consumed as part of a normal human diet. These additional substrates may include non-digestible oligosaccharides, which are preferentially fermented by some gut bacteria before the proteins are utilised, and thus affect the composition of the gut microbiota. Therefore, using less pure proteins, as were used here (75–81%), is more reflective of normal high-protein diets and will generate results with higher translational potential than using ultra-pure protein substrates [9, 62]. Furthermore, hydrolysed proteins are absorbed absolutely in the upper intestinal tract [63]. Consequently, due to increased digestibility, it is highly unlikely that hydrolysed proteins reach the human colon where the majority of bacterial fermentation of dietary proteins occurs. This could, in part, explain the limited translation to *in-vivo* systems where increased dietary casein hydrolysate did not result in significant shifts in bacterial populations in human stools [9].

Although limited in number, the majority of published studies which explore the impact of increased dietary protein on microbiota composition and/or metabolic output do so by using protein derived from single sources [62, 9, 64–66]. However, here we demonstrate that bacterial fermentation of dietary proteins from different sources had differential impacts on colonic bacterial populations and metabolic end-products in vitro. The basis of this probably arises from disparities in the proteolytic capacity of different bacterial groups. For example, bacteria which secrete different proteases and peptidases, including specific species of *Bacteroides, Clostridium* and *Fusobacterium* [4, 67], have growth advantages over other bacterial groups in relation to availability of proteins from different sources. This is a result of varying capacities to cleave exogenous proteins into constituent amino acids, which are utilised more efficiently than larger peptides [68–73]. Subsequently, proteins from different sources are preferentially utilised by different proteolytic groups and thus differential population expansion would occur, as we observed. Linked to this, each of the tested proteins had unique amino acid compositions which may also provide growth advantages to bacterial groups that preferentially utilise specific amino acids [74, 75]. For example, when screened for amino acid content, *Staphylococcus aureus* had significantly higher levels of alanine than other Gram-positive bacteria. These bacteria can eithersynthesise alanine *de novo*, or have increased capacity to obtain alanine from extracellular sources [74]. Since all the proteins used in our trial were non-hydrolyzed, bacteria with lower proteolytic and higher saccharolytic capacity, such as *Roseburia*, were likely to have preferentially utilised any residual carbohydrates present in the protein additives [76]. This could explain why total numbers of *Roseburia* were significantly higher than controls in the non-animal proteins which are likely to contain additional fermentable carbohydrate compared to the animal proteins [4]. Since increased dietary protein is rarely a result of the consumption of an ultra-pure hydrolysed protein from a single source, our results better reflect the effects of high-protein diets on the composition and metabolic output of healthy human gut microbiotas.

Variation in amino acid compositions between the proteins assessed is highly likely to have contributed to the observed differential production of nitrogenous metabolites. The deamination of all three aromatic amino acids (phenylalanine, tyrosine and tryptophan) results in the production of phenol [77]. Similarly high phenylalanine and tryptophan content of milk, whey, soya and mycoprotein [78, 79] resulted in microbial fermentation of these proteins, producing significantly more phenol than fermentation of inulin. Furthermore, decarboxylation of tyrosine results in *p*-cresol production; since mycoprotein typically has low levels of tyrosine, it might be expected that it would produce less *p*-cresol than milk, fish and whey proteins which have relatively high levels of tyrosine [79]. However, we found that mycoprotein fermentation produced the highest concentration of *p*-cresol. While the reason for this is currently unclear, it could be due to the different changes in microbial composition between the proteins leading to elevated levels of *p*-cresol producers under mycoprotein conditions. Both phenol and *p*-cresol have been shown to increase permeability of colonocyte monolayers in vitro [21]. Taken together, our results demonstrate that the source of dietary protein should be taken into account when exploring the impact of high protein diets on microbiota composition and subsequent metabolic activity in relation to gut barrier functionality.

Our results are consistent with the growing body of evidence supporting sexual dimorphism in gut microbiotas [26, 80–85]. As previously noted, female gut microbiotas are often more diverse and have higher overall cell counts than male microbiotas [81, 85, 86]. However, the bacterial species reported to be different between the sexes is inconsistent. For example, males have been reported to have a greater abundance of the *Bacteroides-Prevotella* genera^18,25,26^, and certain species within the *Bacteroides* genus, than females [87], whilst, females have been found to have significantly more lactic acid producing bacteria than males [24]. Although we did observe higher overall bacterial cell counts in females, our study found no differences in the *Bacteroidetes-Prevotellaceae* (BAC) or *Lactobacillus-Enterococcus* (LAB) groups between the sexes at baseline. These discrepancies suggest there are as yet unknown caveats perhaps age could play a key role. Alternatively, our FISHf*low* methodology fully quantifies functional groups while deeper sequencing techniques may well have identified more subtle differences between the sexes at baseline. Additionally, reports of sex-based differences in gut microbiotas are often based on human trials where many additional influential factors are present. For example, bile acids [88] and components of immunity [89, 90], which have considerable impacts on gut microbiotas and are sexually dimorphic, but were absent from our in vitro models [91–93]. Regardless of this, we did observe significantly lower levels of *Clostridium* cluster IX in females than males, which is consistent with previous reports [81, 85, 86]. Interestingly, we report protein × sex interactions regarding changes in microbiota composition in response to increased fermentation of proteins from different sources. Here, *Clostridium* cluster IX (PRO) and *Lactobacillus* spp. (LAB) were present in greater numbers in males following fermentation of mycoprotein and whey protein than in females. Diet-dependent sex differences have previously been reported in animals and humans [82, 84, 94–96], with human males, in general, being more susceptible to dietary-driven changes in microbiota composition than females [82]. However, the underlying mechanisms have yet to be elucidated. During fermentation, differences in microbiotas may be exacerbated by dietary substrate availability, which could impact on bacterial metabolic activity. Although there were only limited sex-dependent microbiota compositional differences in response to fermentation of proteins from different sources, we did observe significant differences between the sexes in microbial metabolite production in response to differential dietary proteins. This is consistent with the metabolic potential of gut microbiotas varying between the sexes. This is of particular interest with regard to the microbial production of phenol in response to dietary fish protein, which was significantly higher in females compared to males. This finding is consistent with females being more susceptible to perturbations in gut barrier function in response to specific stimuli^[17]^. However, we also observed increases in propionate production by the gut microbiota in females in response to fish protein fermentation. Previously, propionate has been demonstrated to ameliorate dextran sodium sulphate-induced colitis in murine models by increasing expression of tight cell junction (TCJ)-associated proteins (e.g. Zona occludin, E-cadherin) [97]. Propionate has also been shown to promote expression of TCJ-associated proteins (claudins and occludins) in healthy pig jejunual mucosa [98].

In conclusion, our results describe strong correlations between increased dietary protein availability, shifts in microbial populations towards more proteolytic phenotypes and subsequent increases in bacterial metabolic end-products linked with increased intestinal permeability In vitro. Our results are consistent with our hypotheses and we demonstrate, for the first time, considerable sex-dependent influences on these interactions. Importantly, we show that both the source of protein is highly influential for microbiota composition and microbial metabolic outcomes. Perhaps caution should be exercised regarding blanket recommendations to increase protein consumption in the wake of the results reported here.

## Electronic supplementary material

Below is the link to the electronic supplementary material.

Supplementary Material 1

Supplementary Material 2

## Data Availability

The data that support the finding of this study are openly available in University of Reading Data Archive at DOI:10.17864/1947.000504.
