# Supplementary figures and images for: Do high-protein diets have the potential to reduce gut barrier function in a sex-dependent manner?
Source: Eur J Nutr. 2024 Apr 25;63(6):2035–54. doi: 10.1007/s00394-024-03407-w (PMC11377480; doi:10.1007/s00394-024-03407-w)

## Slide 1
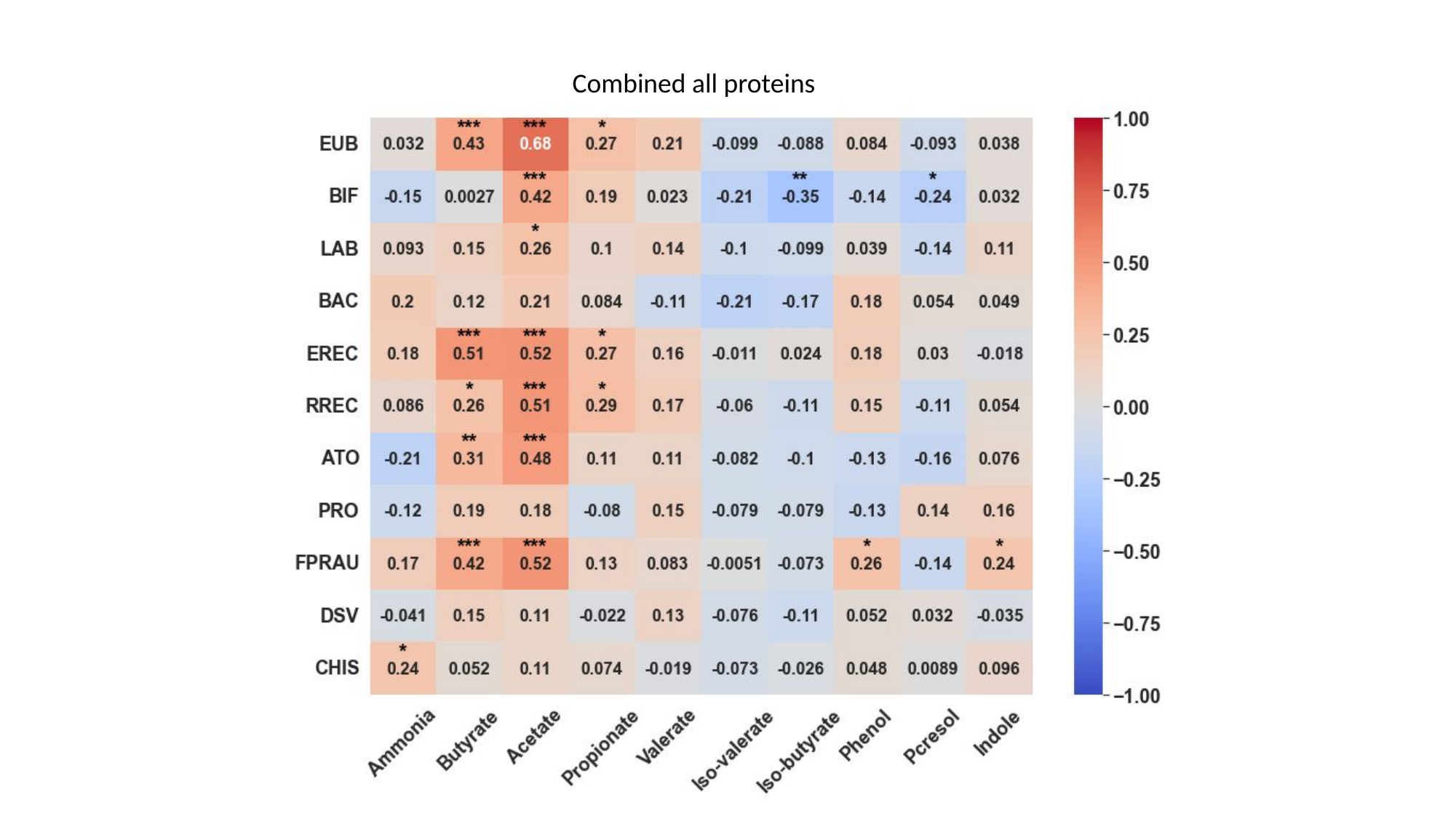

Combined all proteins

## Slide 2
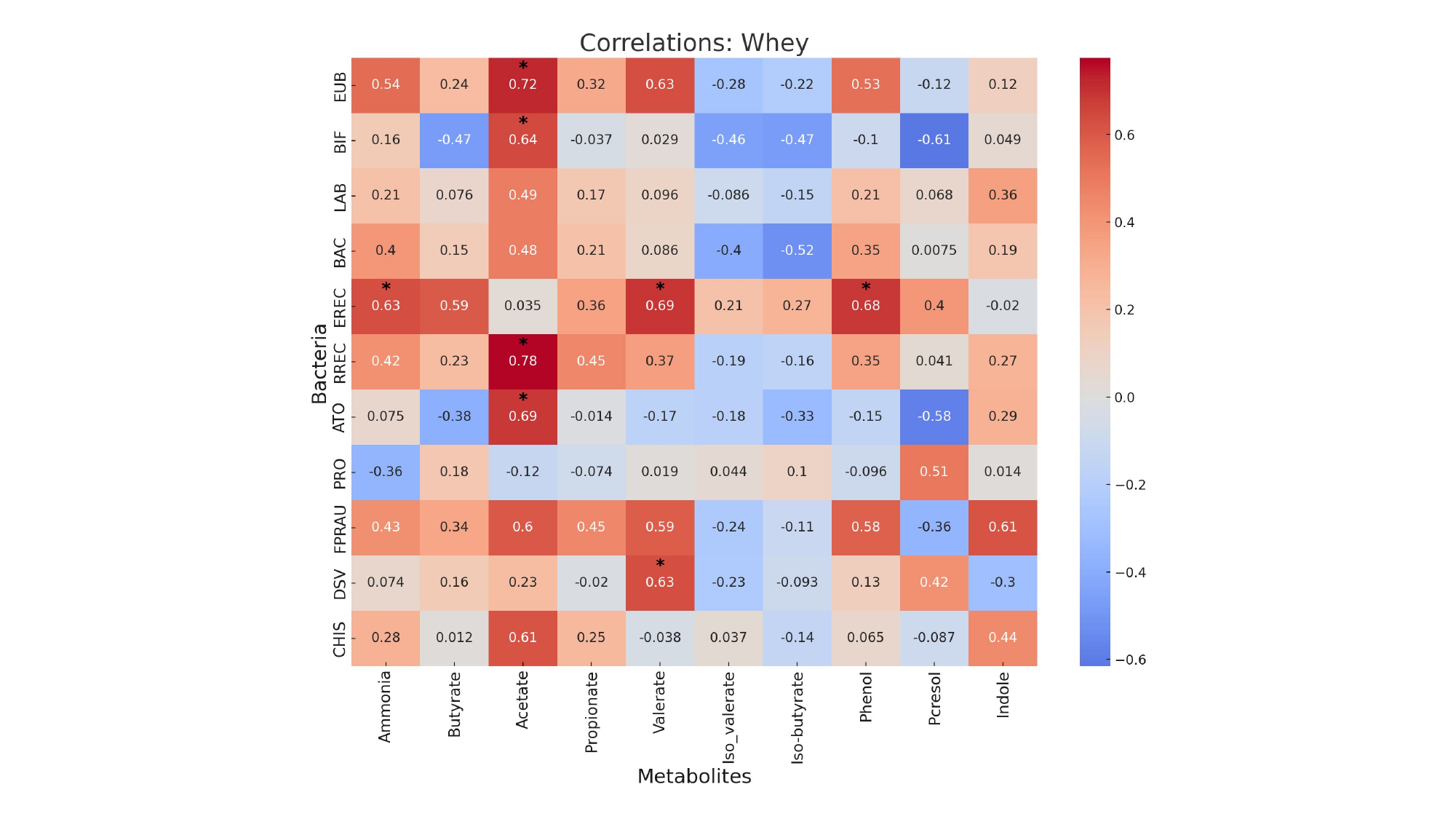

## Slide 3
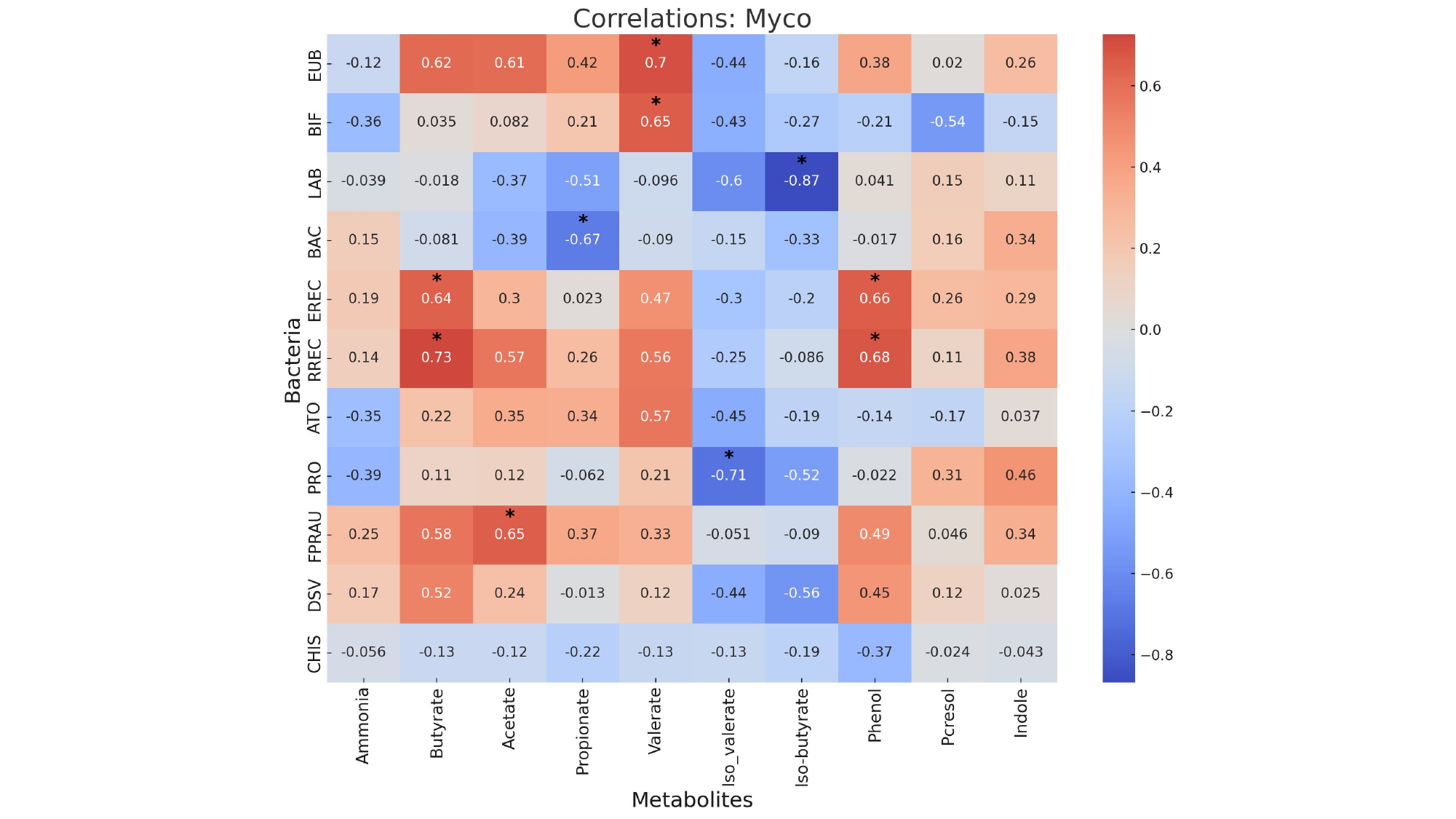

## Slide 4
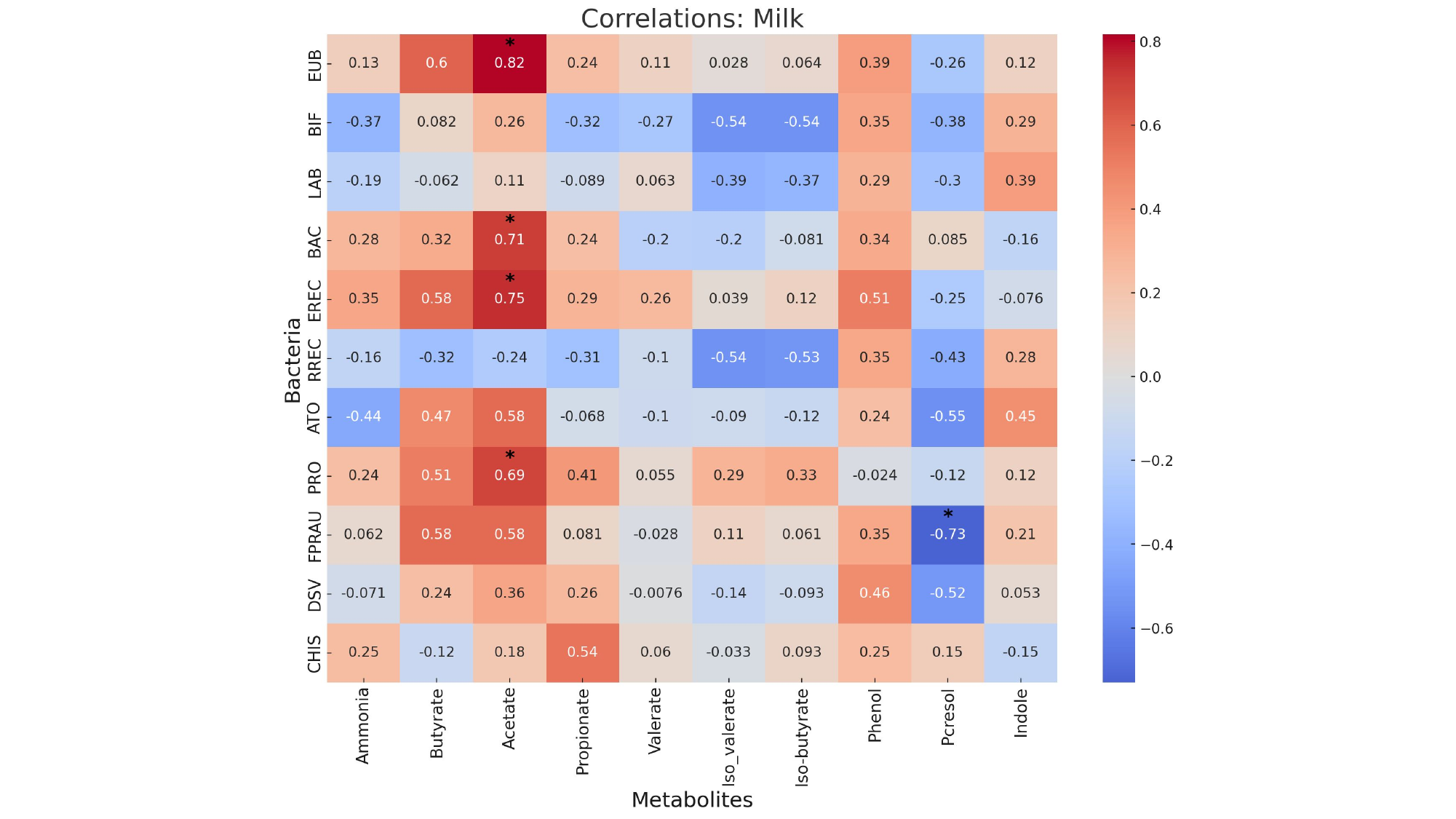

## Slide 5
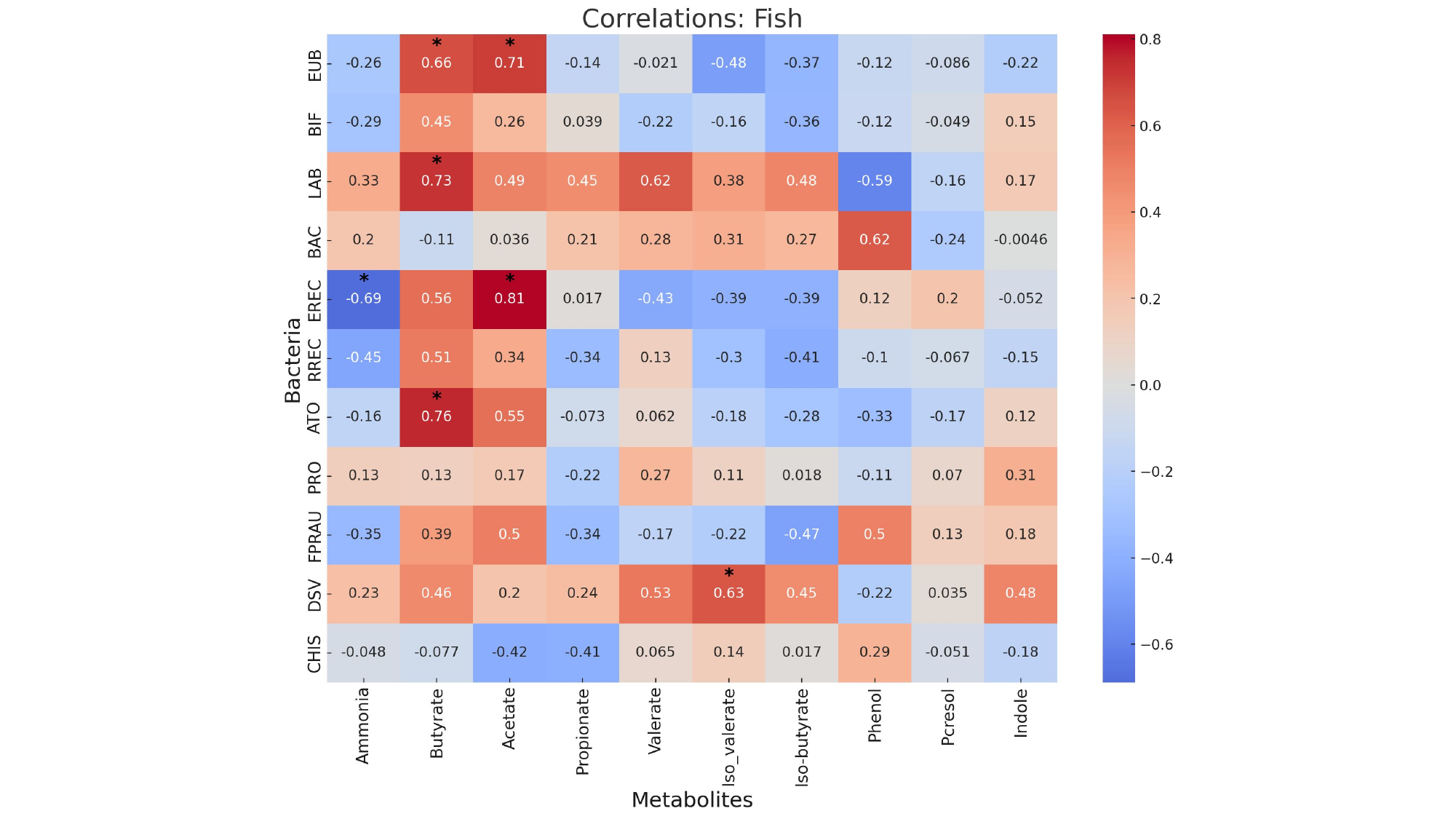

## Slide 6
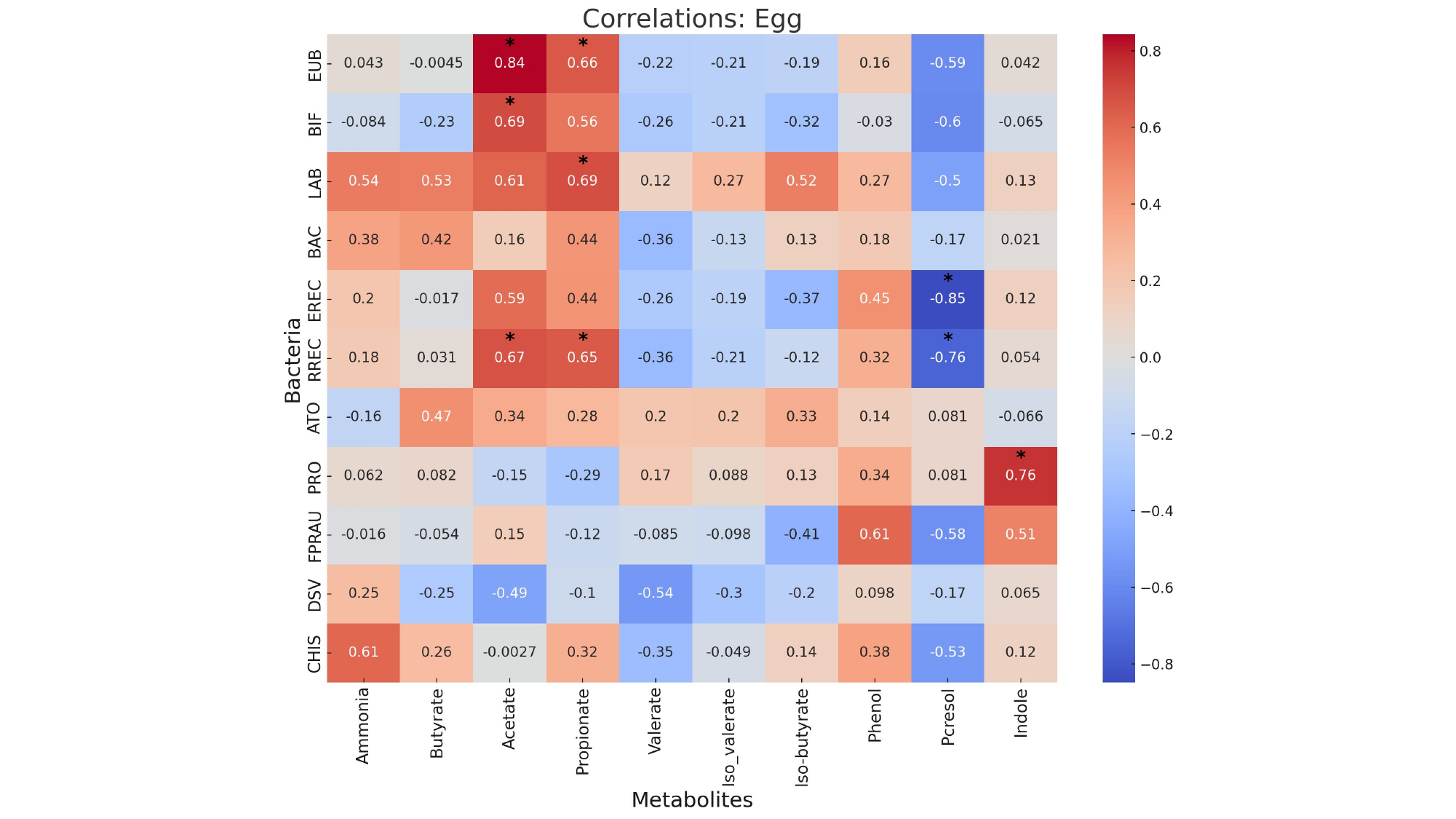

## Slide 7
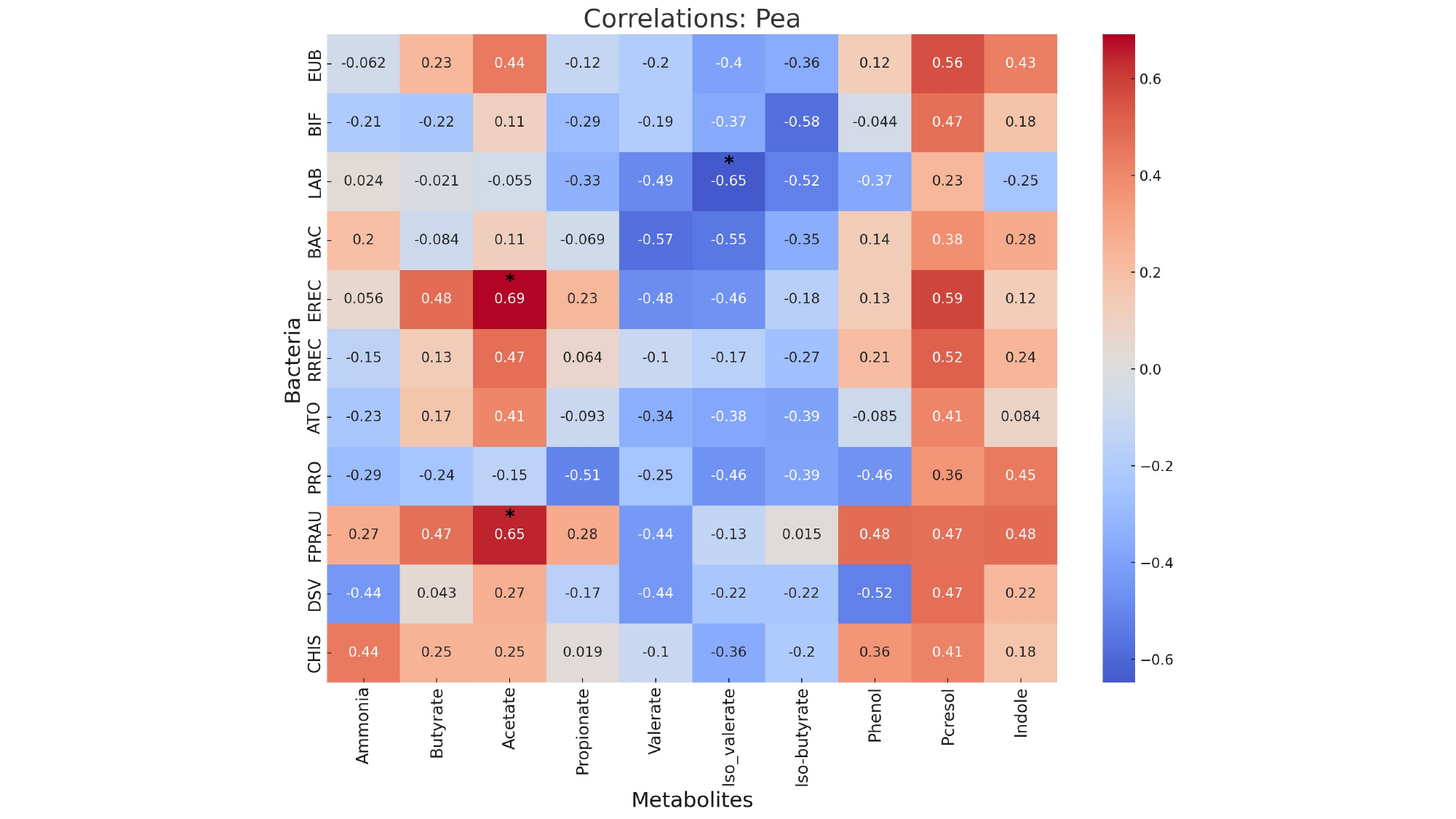

## Slide 8
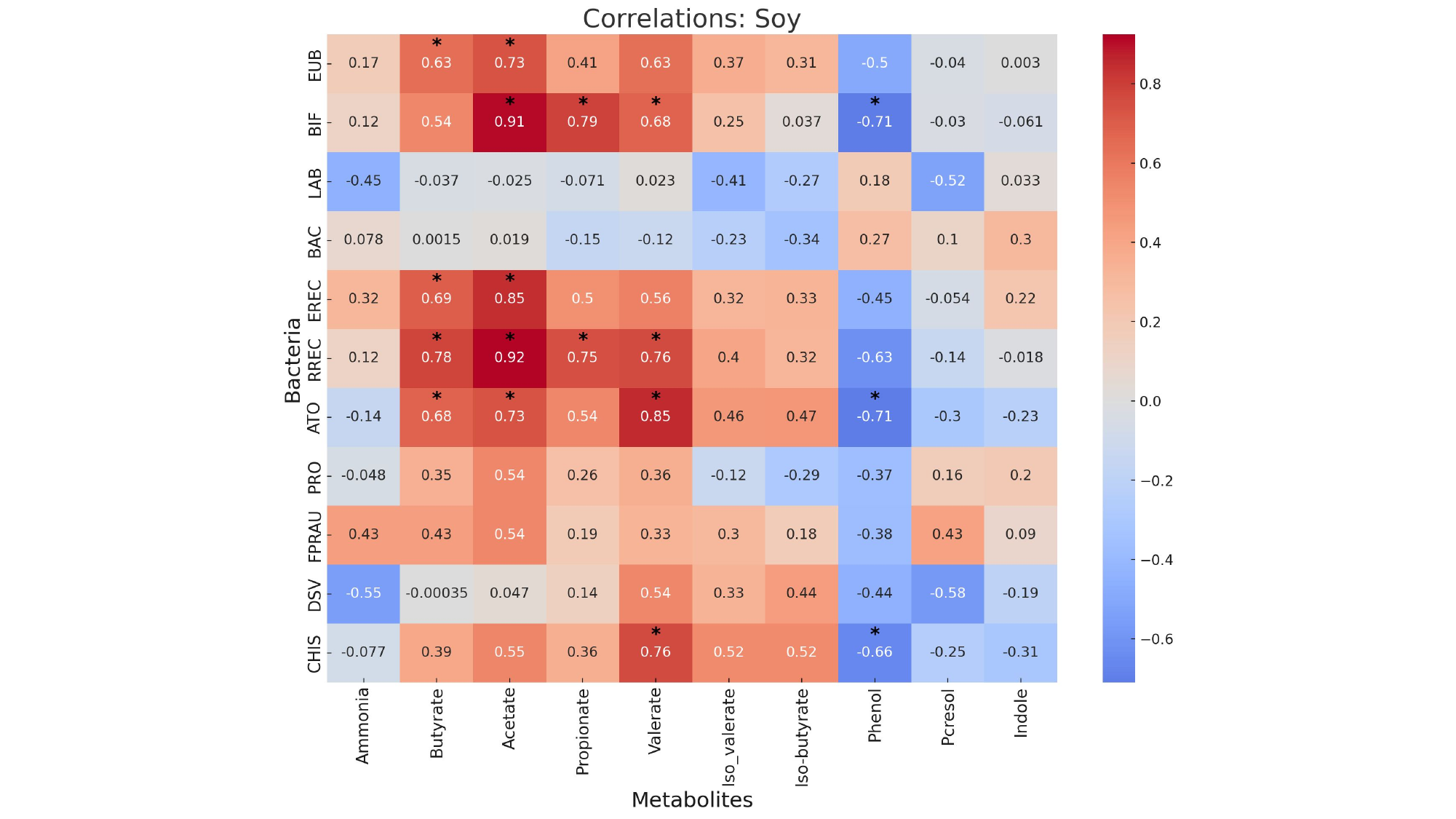

Supplement: Supplementary file 2 — Supplementary Material 2 [file 394_2024_3407_MOESM2_ESM.pptx]
